# The Maturation of the International Health Crisis Response: The Polish Typhus Epidemic of 1916–1923 Compared to the African Ebola Virus Disease Epidemic of 2013–2016: Part I, the Polish Epidemic

**DOI:** 10.3390/epidemiologia5040051

**Published:** 2024-12-09

**Authors:** Gregory M. Anstead

**Affiliations:** 1 Division of Infectious Diseases, Medical Service, South Texas Veterans Healthcare System, 7400 Merton Minter Blvd, San Antonio, TX 78229, USA; anstead@uthscsa.edu; 2 Division of Infectious Diseases, Department of Medicine, University of Texas Health San Antonio, 7703 Floyd Curl Drive, San Antonio, TX 78229, USA

**Keywords:** epidemic typhus, relapsing fever, Poland, international health, *Rickettsia prowazekii*, human body louse, Ebola virus disease

## Abstract

Poland suffered an epidemic of louse-borne typhus from 1916–1923, with 400,000 cases and more than 130,000 deaths. The causative factors were depressed economic conditions and a refugee crisis that engulfed Poland after World War I. The recognition of the epidemic in 1919 stimulated the creation of the League of Red Cross Societies (LRCS). However, the LCRS had limited resources, and the Poles requested help from other governments and the League of Nations (LoN). The United States sent the American–Polish Relief Expedition to conduct delousing. However, the Polish–Soviet War of 1920 disrupted typhus control and exacerbated the refugee situation. The LoN belatedly organized an underfunded Epidemic Commission. The LCRS sent a research team that did groundbreaking work on the pathology of typhus. Into 1921, the epidemic continued, driven by refugees from typhus-stricken Russia. By 1924, typhus cases were finally approaching pre-World War I levels. Multiple factors lead to the amelioration of the epidemic. The repatriation of prisoners of war and displaced civilians had concluded by 1923. Also, there had been a steady influx of sanitary, food, economic, and medical aid from various organizations into Poland since 1919. Administratively, within Poland, the anti-typhus campaign was also conducted more effectively by the Extraordinary Epidemic Commissariat.

## 1. Introduction

In 2014, global attention was transfixed on an escalating epidemic of Ebola Virus Disease (EVD) transpiring in the West African nations of Guinea, Sierra Leone, and Liberia. By 30 June 2014, there had been 759 cases, with 467 deaths [1]. At that time, the number of cases in this latest epidemic far exceeded the previous worse EBV outbreak (Uganda, 2000), in which 425 people were infected [2]. In July 2014, 11 West African ministers of health met with representatives from the World Health Organization (WHO) and various international aid organizations to formulate a strategy to arrest the epidemic [3]. On 28 August 2014, the WHO released its Global Response Roadmap, which included: establishing treatment centers with infection prevention and control capability; referral processes for primary care facilities; case diagnoses by certified laboratories; enhanced surveillance; supervised burials; control of borders and travel; and community engagement in contact tracing and risk mitigation [4].

In September 2014, Médecins Sans Frontières (MSF) urged all nations with epidemic response capability to intervene in the EVD outbreak, which had already caused 5900 deaths. MSF declared that without the assistance of foreign governments, the United Nations and the currently involved nongovernmental organizations were unable to fully implement the WHO Roadmap [5].

Between December 2013 and April 2016, there were 28,616 reported cases and 11,310 deaths from Ebola virus disease (EVD) in West Africa [6]. Twenty-eight months after its origination, the multi-pronged approach by multiple agencies successfully terminated the largest recorded EVD epidemic. Nevertheless, during and after the outbreak there were several critiques that the delay in the international response contributed to the human cost and its economic disruption.

Almost 1 century earlier, an epidemic of louse-borne (epidemic) typhus ravaged Poland from 1916–1923, with over 400,000 cases and more than 140,000 deaths [7]. The principal manifestations of epidemic typhus are fever, headache, rash, altered mental status, peripheral gangrene, myocarditis, and secondary infections; in the pre-antibiotic era it carried a mortality rate of 20–60%. The causative organism is the bacterium *Rickettsia prowasekii* [8]. Persons who survive infection with *R. prowazekii* are immune to re-infection. However, *R. prowasekii* can lay dormant for years in the adipose tissue of recovered patients. Physiologic stress, such as malnutrition, may cause this latent infection to reactivate, a condition known as Brill–Zinsser disease, and these patients become potentially infectious once again (although the recrudescent infection is less severe) [9].

Outbreaks of epidemic typhus are often accompanied by another louse-borne infection, relapsing fever, due to the bacterium *Borrelia recurrentis*. Relapsing fever presents with fever, headache, body aches, and fatigue, and carries a 10% mortality rate. Death may ensue from myocarditis, acute respiratory distress syndrome, cerebral or gastrointestinal bleeding, splenic rupture, or hepatic failure [10]. Poland also suffered 67,578 cases of relapsing fever from 1919–1923 [11].

Despite the markedly different etiologies and modes of transmission of EVD and typhus, there are striking parallels between these two epidemics. Both occurred in settings of impoverishered populations of low literacy with grossly deficient medical infrastructures. Both are contagious, carry a high mortality rate, and obligate health workers to don protective gear. As in epidemic typhus, once the epidemic of EVD is ignited, humans are only the reservoir. For both maladies, handling of the deceased and their clothing represents a contagion risk. Control of human transit were paramount for the containment of the two infections. For EVD in 2013–2016 and for epidemic typhus in 1916–1923, there were no established vaccines and no specific treatments. Thus, the control of the dissemination of both infections depended on case detection, isolation of infected individuals, and the erection of sanitary cordons. Both epidemics generated international attention and required a multinational effort for suppression. It is instructive to examine the response to these two epidemics, nearly a century apart, and assess how the international health crisis response apparatus has evolved.

In Part I of this two-part series, the course of the Polish typhus epidemic will be addressed in detail. As will be seen, the international response was marred by indecision, geopolitical animosities, factional self-interests, and war. However, after 7 years, a conglomeration of factors and events in multiple countries—philanthropic, political, economic, historical—facilitated the resolution of the Polish typhus epidemic. Part II will focus on the African Ebola epidemic of 2013–2016. Although, the initial international response was considered to be sluggish, ultimately the EVD epidemic brought about rapid advances in disease surveillance, diagnostics, antiviral therapeutics, and vaccinology. The COVID-19 pandemic erupted in the spring of 2020. On the one hand, the COVID-19 pandemic energized the global biomedical research community to develop vaccines, therapeutics, and diagnostics like no other problem ever before. However, politics, both domestic and international, once again thwarted a coordinated response to an even greater infectious diseases threat.

## 2. The Polish Typhus Epidemic: Recognition and Initial International Response

**Figure A1:**
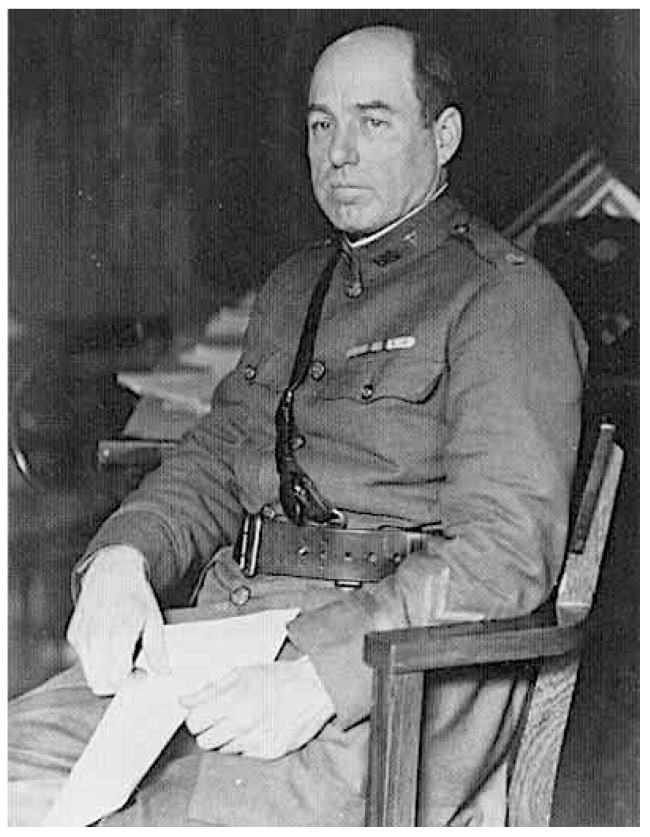
Richard P. Strong, Medical Director of the League of Red Cross Societies [140].

After World War I, Henry P. Davison, Sr., an American banker who had chaired the War Council of the American Red Cross (ARC), championed the creation of an organization to coordinate the work of national Red Cross societies. Davison had witnessed the immense popular support for the ARC during the war, and it was his vision that this fervor could be harnessed for the advancement of global health. In April 1919, Davison organized the Medical Conference of Cannes, which assembled representatives from 24 national Red Cross societies and scientific and public health experts from around the globe [12]. The original program addressed sexually transmitted infections, malaria, preventative medicine, and child welfare. After the meeting adjourned, an emergency session was called by Davison, due to an alarming telegram that he had received from the ARC declaring that Europe was now facing the most calamitous disease threat since the Second Pandemic of Plague—the dread scythe of louse-borne disease was sweeping across Eastern Europe. At the conclusion of the emergency session, Davison addressed a telegram to the “Big Four” delegates of the Inter-Allied Peace Conference (Georges Clemenceau, Woodrow Wilson, Lloyd George, Vittorio Orlando) warning about the rapid extension of typhus into Eastern Europe and appealing to the Allied governments to provide the necessary aid to suppress the epidemic [13]. A committee of experts was convened, comprised of Richard Strong (Chairman; former director of the International Sanitary Commission to Serbia and the American Trench Fever Commission) (Appendix A, Figure A1), Aldo Castellani (pathologist who had served on sanitary commissions in Serbia and Macedonia), Col Frederick Russell (physician, US Army Medical Corps), Col S. Lyle Cummins (pathologist, Royal Army Medical College), and René Legroux (microbiologist, Institut Pasteur). In a speech, Strong emphasized that a centralized “Bureau of Hygiene” was preferable to the uncoordinated actions of individual Red Cross societies or overwhelmed local authorities [13]. The concept of a “Bureau of Hygiene” became embodied in the League of Red Cross Societies (LRCS). By 5 May 1919, the LRCS Articles of Association had been drafted and the organization’s stated mission was the global prevention and alleviation of disease [12]. However, from its inception, the LRCS was embroiled in controversy. The representatives of the Swiss-founded International Committee of the Red Cross (ICRC) believed the appropriate role of the Red Cross was only to provide aid to captives during wartime. However, supporters of the LRCS proposed that the pervasive effects of modern warfare dictated that all medical and material consequences for both civilians and military personnel fell within the purview of an international relief organization [14].

Typhus had long been endemic in Poland. In Russian Poland before World War I, there were about 6000–7000 cases per year, in a population of about 12.8 million; in Galicia (Polish territory incorporated into Austria-Hungary), cases had been declining prior to World War I [15]. However, in 1915, cases of typhus significantly increased, concurrently with the maneuvers of Czar Nicholas’ Army [16]; likewise, in the areas of Poland occupied by the Central Powers, the number of cases swelled to over 12,000 [15]. The German civil government of German-occupied Russian Poland was established in January 1915, and the control of communicable diseases was given a high priority. The chief German medical officer in occupied Poland, Dr. Gottfried Frey, was assisted by 50 district medical officers. Medical parasitologist Erich Martini from the Institute of Ship and Tropical Diseases in Hamburg served as the typhus commissar. The Germans considered the Poles, and especially Polish Jews, to be inherently filthy and disease-ridden. Thus, the German authorities launched an educational campaign to promote hygienic behaviors in occupied Poland [17]. The Germans improved sanitation systems, water supplies, and municipal baths and constructed showers. Hundreds of steam disinfection units and formalin chambers were imported from Germany for the delousing of clothing. Between July 1916 and November 1918, 3.5 million Polish civilians were deloused, and 480,000 dwellings were fumigated. Three hundred quarantine stations and isolation hospitals were established [7,18,19]. Frey also set up a hygiene institute in Łódź and laboratories in Plock and Ostroleka. German bacteriologist Richard Otto, from Berlin’s Koch Institute, was posted to Wilno (present-day Vilnius, Lithuania), where he supervised laboratory and hospital facilities and directed 30 disinfection squads that deloused 200,000 persons in 1 year [7]. However, the anti-typhus measures instituted by the Germans in Poland were often coercive. The disinfection squads forcibly deloused civilians, searched their homes, boarded up the houses of typhus victims, and evacuated whole neighborhoods. Persons suspected of having typhus were detained, and their contacts were quarantined for 14 days [7,17]. For the Germans, the vigorous anti-typhus campaign in Poland came at a high price; 10 of the 50 physicians stationed there succumbed to the infection [17].

Despite the massive delousing efforts of the Germans, the number of typhus cases increased in the areas of Poland occupied by the Central Powers, from 34,538 in 1916 to 47,840 in 1917. Typhus also struck Warsaw, with 25,000 cases registered from January 1917 to July 1918 in a population of 700,000. The case fatality rate in various areas of Poland ranged from 7–21% [15]. In 1917, Poland suffered 45,000 typhus cases; in 1918, this number swelled to 97,000 [16].

From the ashes of World War I, Poland was resurrected in late 1918, reassembled from fragments of Russia, Germany, and Austria–Hungary. The new republic was saddled with formidable impediments. After 120 years of partition, the new Poland was a patchwork of areas with differently organized administrations, currencies, legal codes, and railroad gauges [20,21]. Few Poles had experience in civil administration, because during the century of foreign occupation most of these positions had been denied to them. The nation had lost a significant portion of its intelligentsia from deportation, emigration, and attrition during World War I [22]. More than half a million Poles had died in battle, and hundreds of thousands had succumbed to malnutrition and disease [23]. After Serbia, Poland had suffered the most materially from World War I, enduring successive waves of invading and retreating armies. The Russians practiced a scorched earth policy in the areas of Poland from which they retreated, resulting in millions of homeless and mass migrations within the country [16]. Also, about 700,000 Poles had fled to Russia to escape the advancing Germans [23]. (For an eyewitness account of the devastation of Eastern Poland in the aftermath of World War I, see Appendix C, Quote 1).

**Figure 1:**
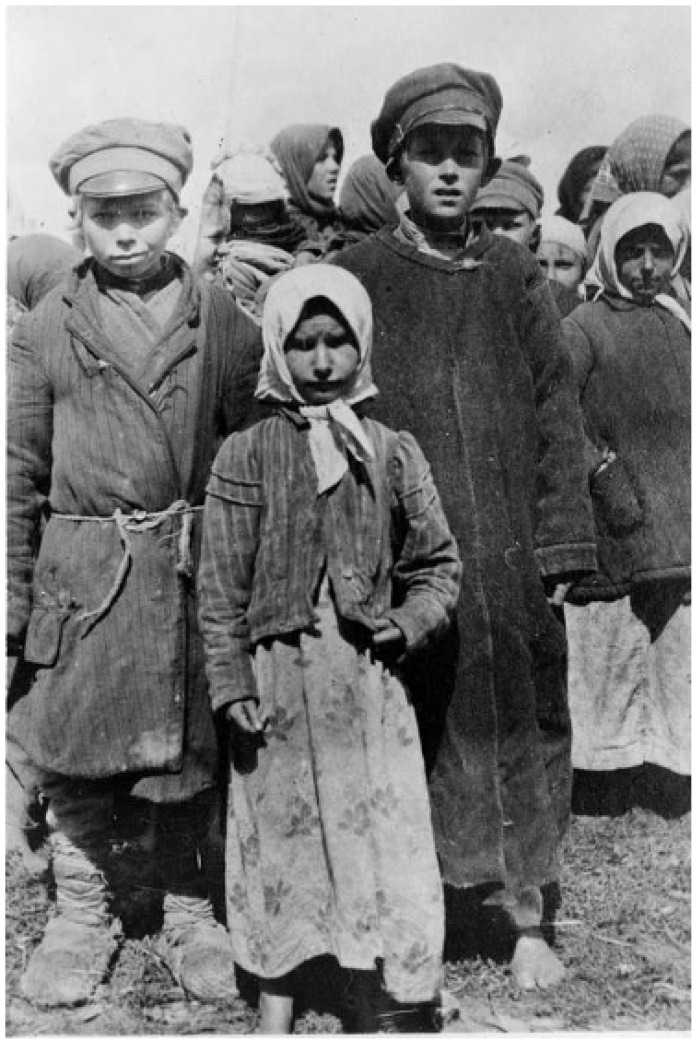
Original Captions: “… undernourished children received at the refugee station at Rowno visited by the Interallied Medical Commission…” “A lair for typhus lice” [26]. Summer 1919.

The new Poland suffered from complete economic collapse, with shortages of food, water, coal, and clothing, and a dearth of medical personnel and resources [15]. Ten million Poles were malnourished [24] (Figure 1). One British aid worker reported that the population was living upon roots, grass, acorns, and heather, and that the only available bread was composed of those ingredients, with about 5% rye flour [21]. Soap was scarce because there were no excess fats for its production [24]. Food shortages resulted from the destruction and confiscation of crops during the war, and a lack of manpower and draft animals to till the fields. In 1919–1920, the acreage under cultivation was only 61% that of 1913–1914. Almost one-third of Polish cattle had been seized by pillaging German and Russian soldiers during the war. There were neither raw materials nor markets, and the factory machinery had been carted off by retreating armies. Factories, such as the renowned textile mills of Łódź, lay dormant, leaving thousands of families destitute [25]. Overall, about 85% of Poland’s industrial workers were now unemployed [23].

In the immediate post-war period, the American Relief Administration (ARA), directed by Herbert Hoover, committed to feeding 2.5 million Poles and delivered: 216,000 tons of flour; 72,000 tons of beans, peas, and rice; 54,000 tons of fats; and 2400 tons of milk. Additional delivered items included sewing machines, clothing, automobiles, tires, tools, and sanitary supplies. The first food shipments arrived in February 1919. The ARA also set up a Transportation Section to revitalize Poland’s decrepit rail system. Locomotives and other rolling stock were sent from Germany as reparations for their wartime plunder. In April, the ARA obtained supplies from the American Expeditionary Force in France for shipment to Poland, which included food, clothing, railroad equipment, and horses [25]. In June 1919, Poland also received a $4 million ($72.6 million in 2024 US dollars) loan from the ARA for child feeding programs and for the purchase of vehicles and sanitary materials (soaps, disinfectants, clothing, etc.) [11]. (For inflation adjustments and currency conversions, see references [27,28], respectively. For subsequent historical monetary amounts, the value in 2024 US dollars is indicated parenthetically). By autumn 1919, the ARA had delivered 751,135 tons of food and 30,000 tons of cotton [29]. In 1919, the ARA fed more than 1.5 million Polish children, which increased to two million in 1920; 10,000 kitchens were established. During the 4 years following the war, the ARA served half a billion meals to hungry Poles [30].

**Figure 2:**
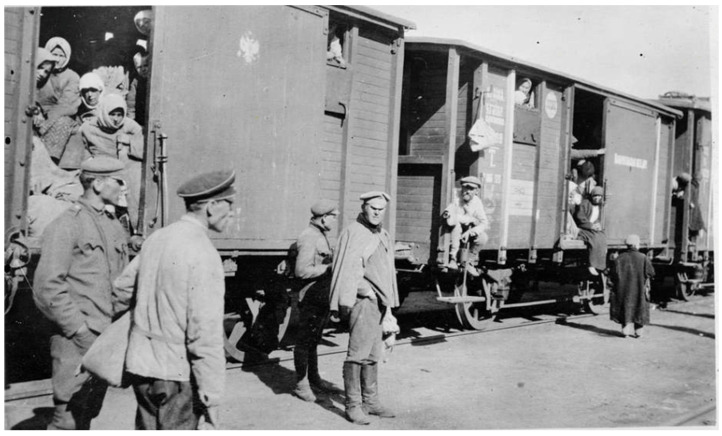
Original caption: “Freight trains crammed with refugees returning to their homes in Poland after having passed through the delousing and disinfecting stations on the eastern border” [39]. 11 October 1919.

Post-war Poland was a vast highway of human migration east and west. Between 1 November 1918 and 1 January 1920, 652,604 prisoners of war and 627,088 emigrants returned from Russia to Poland [31,32] (Figure 2). Many of the refugees reaching Poland had traveled from Siberia for months and arrived in a destitute and debilitated condition [33,34]. The number of refugees within the war-torn areas of Poland was estimated to be 200,000. In addition, some 554,000 Russian prisoners of war were returning eastwards across Poland from Germany and Austria [15]. An estimated 2.9 million refugees entered Poland in the first 14 months following World War I [35]. Former prisoners of war, to evade re-induction into the military, often selected desolate routes to make their way back to their villages to avoid police checkpoints, thereby missing health inspections, and when they arrived in their hometowns, they established new foci of typhus [36,37]. Under such conditions of economic deprivation, chaos, and human migration, typhus blossomed. In 1918, there were 122,000 cases of typhus recorded in Poland; the number nearly doubled to 231,000 in 1919, with about 20,000 deaths [15,38].

**Figure A2:**
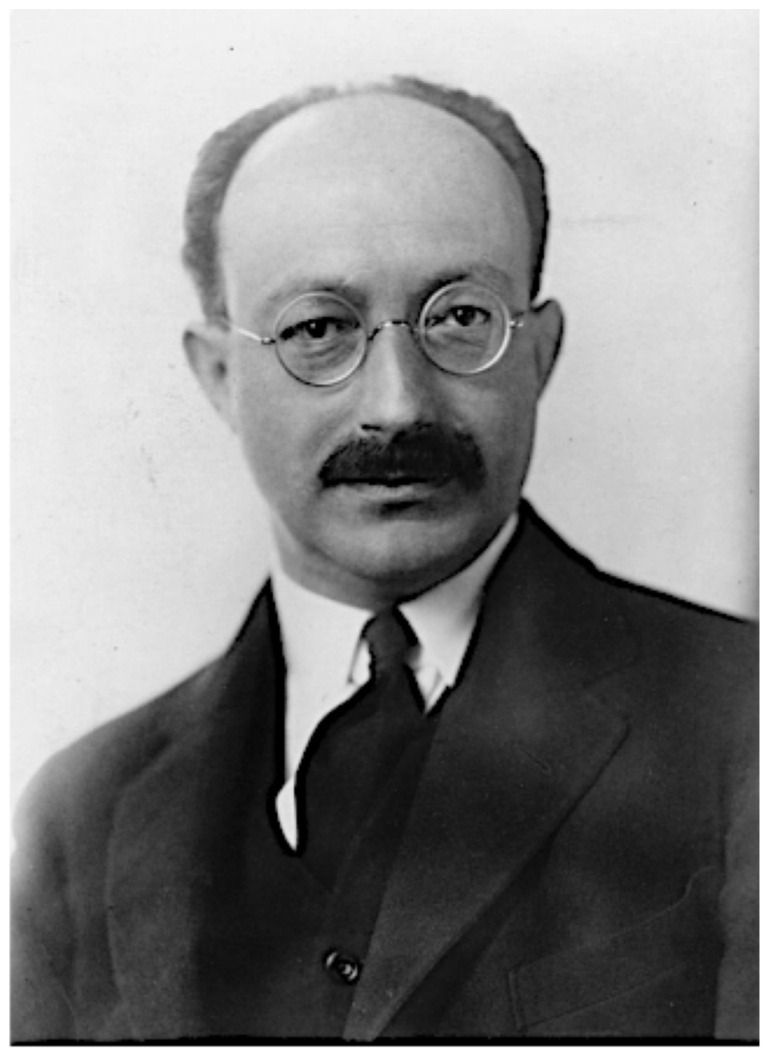
Polish physician Ludwik Rajchman, founder of the National Institute of Hygiene and Public Health and a commissioner of the Epidemic Commission of the League of Nations [22].

Initially, the fledgling Polish government implemented a few feeble steps to quell the epidemic, but the situation was overwhelming. Poland was severely deficient in medical personnel, equipment, and facilities; it had lost 4000 physicians in the war and only had about 8% of the number that was needed to meet the needs of the country [40]. At war’s end, Germany withdrew much of its medical and sanitary infrastructure from Poland [18,40]. Polish bacteriologist Ludwik Rajchman founded the National Institute of Hygiene and Public Health (NIHPH) soon after his return to Poland in November 1918 (Appendix A, Figure A2). The missions of the Institute were research on infectious diseases, laboratory diagnostics, and the production of vaccines and antisera [41]. With colleague Czeslaw Wroczynski, Rajchman pursued the departing Germans to purchase sanitary supplies and laboratory equipment that they were removing from the country. The NIHPH dispatched physicians to the areas with the highest disease prevalence, and medical students were recruited for the sanitary inspection of railway passengers [42]. In response to the competition from the LRCS, the ICRC deviated from its prior 60-year history as a strictly military support organization and began to assist civilian victims of disease and famine [19]. Frédéric Ferrière of the ICRC toured Poland in early 1919 and reported: “There is no soap at all, no linen, and the unfortunate sufferers, wrapped in their verminous rags and goatskins crawling with lice, carry the infection from place to place…” [37].

Post-war Poland was a seething cauldron of pestilence, gripped by outbreaks of relapsing fever, dysentery, typhoid, diphtheria, pertussis, smallpox, scarlet fever, and sexually transmitted diseases in addition to typhus. Tuberculosis claimed the lives of nine of every thousand Poles. The largest cities (Warsaw, Łódź, Lvov, Cracow, and Wilno) were especially tormented by infectious diseases. Rajchman endeavored to establish a network of disease surveillance units throughout the country, but his efforts were hampered by a lack of facilities and trained personnel [11,22].

Meanwhile, the international community was slowly responding to Poland’s plight. In March 1919, the ICRC set up the Central Epidemiological Bureau for Eastern Europe, which included delegates from Poland [43]. In the spring of 1919, the ARC sent a contingent of about 150 relief workers, who distributed clothing, provided medical care, and helped establish preventive measures against typhus [32]. On 15 May 1919, Arthur Balfour, President of the LoN Council and Britain’s former Foreign Secretary, appealed to the LRCS for additional aid to mitigate the European typhus crisis; he had failed in convincing his own government to aid the Poles. Officials at the British Treasury opposed any financial support for Poland, except for loans. The British Cabinet’s LoN Committee advocated for a grant of a grant of £50,000 ($4 million), but only if funds were also committed by the USA, France, Holland, and Spain [44].

In June 1919, Poland appealed to the Western powers for assistance. Hoover thereby introduced a resolution to the Supreme Economic Council (a body created in 1919 to coordinate resources to facilitate post-War economic recovery) to aid Poland in its battle against typhus. With Wilson’s support, Hoover arranged to send laundries, linens, beds, soap, motorised bathing facilities, underclothes, and ambulances to Poland [25,42].

At a conference in Paris in June 1919, the Polish Minister of Health Tomasz Janiszewski requested that the LRCS assist Poland. Two proposals were floated: (1) the LRCS should manage a cordon sanitaire (quarantine line) along Poland’s eastern border, and (2) the LRCS should assist the Polish government in finding suitable personnel to carry out a typhus control program [32]. Representatives of the LRCS retorted that it was not within its purview to staff a quarantine line, but they did make a request to the USA to sell or donate the necessary supplies and arrange for sanitary personnel to be dispatched to Poland [12].

In July 1919, the Allied Supreme Council requested that the British, French, and Italian LRCS representatives petition their governments to take measures against typhus in Eastern Europe. The LRCS started organizing a multi-national planning conference to determine the approach to typhus control in Poland, but, due to lingering animosities, Germany was not invited to participate. This was a serious omission because of Germany’s obvious strategic geographic position, its self-interest in typhus control, and its established proficiency in louse control based on expertise acquired during World War I. Due to the exclusion of Germany, the ICRC refused to join in either the planning conference or the campaign against typhus itself, because rejecting any country was against its founding principles. The planning initiative of the LRCS thereby collapsed [19].

**Figure 3:**
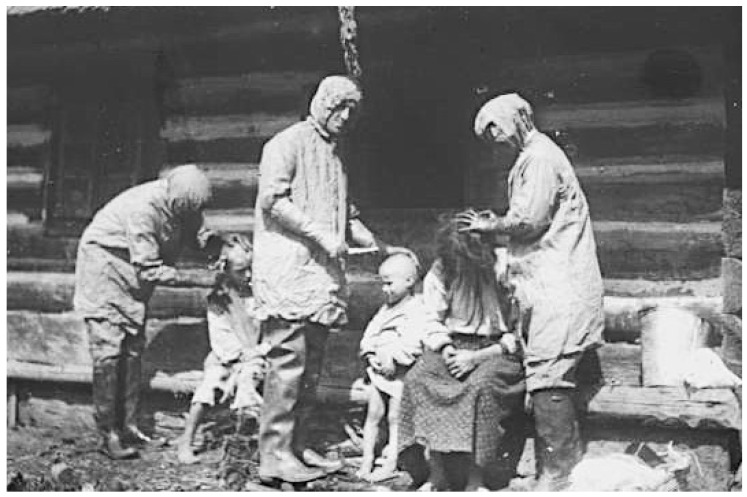
Original caption: “[Quaker] delousing unit checking members of family for lice” [47]. Poland, 1919.

**Figure A3:**
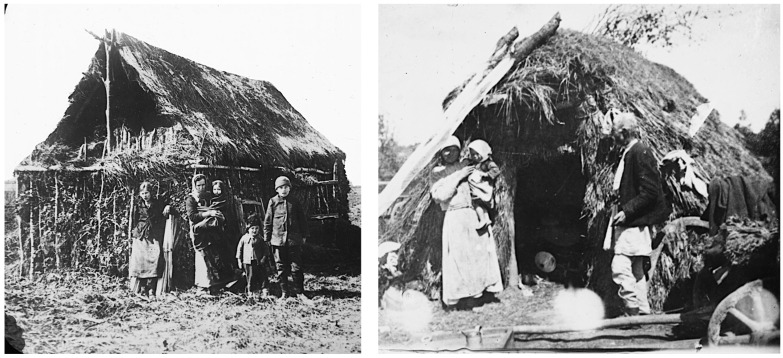
**Left**: “Widow and four children outside of shabby hut” [141]; **Right**: “Members of family outside of very shabby, poverty-stricken dwelling” [142]. Poland, 1919.

In summer 1919, in response to an appeal from the Polish Ministry of Health, the British Society of Friends (Quakers) sent a medical unit, directed by Dr. Edward W. Goodall, an infectious diseases specialist from the Eastern Fever Hospital in London [15,32]. The Quaker mission also disinfested persons and their dwellings, operated orphanages, and improved agricultural production by supplying seeds, farm implements, and horses [45]. Many of the refugees had been subsisting on a diet of black or acorn bread, wild herbs, and cabbage. The Quakers distributed “famine packs” of beef extract, condensed milk, biscuits, canned sardines, and baby formula. The relief workers witnessed families living in crowded, dilapidated houses (see Appendix A, Figure A3) and “refugees camping in their bough shelters in the forests, drainage ditches, or in former Army dugouts, looking as though they were covered in a white moving curtain of lice… Members of the [Quakers] mission’s typhus units stood by week after week in their delousing stations clad in black rubber boots and silver mackintoshes… They were armed with clippers and razors and bottles of disinfectants. They lived in a world of steam, surrounded by naked patients, some skeletally thin, others with swollen stomachs that masked starvation. All the hair had to be shaved off the patients’ heads and bodies to get rid of the lice which swarmed so thickly that at times the horrible creatures had to be removed with the blade of a knife” (Figure 3) [46].

By mid-1919, Polish health authorities had implemented a vigorous response to the crisis. They organized 44 mobile fever hospitals with 2200 beds, 103 community hospitals with 4400 beds, and 35 mobile disinfection units. Twenty-three infectious diseases physicians collaborated with district physicians. Nevertheless, formidable barriers to further progress remained: poorly equipped hospitals and shortages of medical personnel, sanitary supplies, drugs, and soap [48]. The prospect of securing sufficient private aid to overcome Poland’s crisis looked bleak. An international conference on the European health situation was convened at the British Ministry of Health in July 1919, but the conferees failed to develop an action plan [14]. In November 1919, David Henderson, Director-General of the LRCS, acknowledged the inability of the organization to control the scourge of typhus without governmental aid [49]. Despite the prior success of voluntary agencies and the International Sanitary Commission in suppressing typhus in Serbia in 1915 [32], the epidemic in post-war Poland presented a much more difficult situation. Poland was much larger than Serbia in both population and land area, and the rampant ongoing typhus epidemic in Russia and the massive migrations from that country created a continual influx of new typhus cases and louse-infested refugees.

For Europe, the potential consequences of an uncontrolled typhus epidemic in Poland were dire; relief officials feared that if the march of typhus was not staunched in Poland, it would penetrate Western Europe [38]. There was some justification for this concern. In 1919, in Holland, there was a typhus epidemic, with 87 fatalities, that occurred from 1 January to 27 May in the poorer sections of Rotterdam. Local hospitals were so overwhelmed that two schools were converted into fever hospitals [50]. The Dutch outbreak was ignited by typhus-infected immigrants and the importation of used clothing infested with louse eggs [31,50,51]. There were 833 cases in Holland from January through April of 1919, compared to only nine from 1911 to 1917. Holland was able to quickly control the epidemic by quarantine and sanitary measures because it was a more developed country that had not suffered the economic devastation of the war [50].

Typhus infiltrated neighboring Slovakia in 1919–1920, resulting in over 1300 cases and 160 deaths [52]. The German border was breached by typhus as well, due to the flux of repatriating German civilians, 150,000 returning prisoners of war, and the outward migration of Russian prisoners of war. By March 1919, there were over a thousand cases in Prussia, with hundreds of deaths. Furthermore, after the 1918 armistice the German army relinquished its Ukrainian territories, precipitating the migration of 120,000 Germans and Jewish refugees through typhus-ridden lands on their westward trek to Germany. In 1920, German public health authorities instituted a cordon sanitaire on its eastern border, which included a chain of bacteriological stations and a hygiene institute in each major city [7]. The German Interior Ministry favored cooperation with Poland and its other neighbors in the anti-typhus campaign. However, the French government opposed any German presence in Poland [19]. German participation in matters of transnational health was obstructed in the early post-war era by its continuing pariah status. Even though typhus raged on its borders for over 3 years, Germany was not invited to participate in an international health conference until 1922 and it was denied admission to the LoN until 1926. Scientists at the various Pasteur Institutes declined to collaborate with their German counterparts [53]. In turn, German scientists also isolated themselves from their European colleagues due to their own resentments [7,19].

There were also concerns that further deterioration of social conditions in Poland might promote the spread of Bolshevism [38]. Winston Churchill went so far as to conflate the spread of Bolshevism with the transmission of typhus across borders [54]. A 1920 headline in an Australian newspaper read: “Parlous Poland. Ravaged by Bolshevik typhus” [55]. Many Poles blamed typhus on the Russians and Polish Jews, who were thought to be sympathetic to the Bolsheviks [41].

## 3. The Work of the American–Polish Relief Expedition and the League of Red Cross Societies

**Figure A4:**
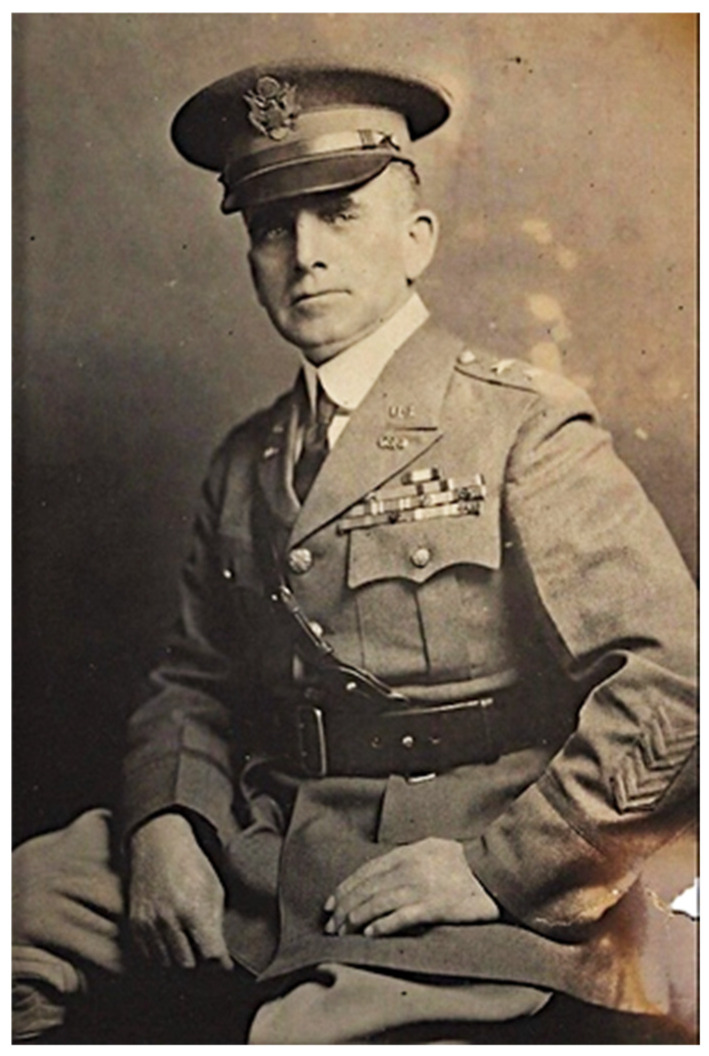
Major General Harry L. Gilchrist, commanding officer of the American Polish Relief Expedition [143].

By March of 1919, several countries had requested medical assistance from the Big Four Allied leaders, but they stalled. On 4 April 1919, Hoover was summoned to a meeting of the Big Four to offer his opinion on how to respond to the typhus emergency. Hoover thought it would be best addressed by the Red Cross societies. However, both the ARC and the LRCS rebuffed him, declaring that the crisis was far beyond their resources. Hoover then convened a meeting in Paris with the ministers of health of several of the involved nations. At that point, Hoover claimed there were an estimated one million cases of typhus in Eastern Europe, with 100,000 deaths weekly [56]. (The number of deaths was certainly an exaggeration). Hoover relayed the information to the Big Four and recommended that the Allies furnish the necessary aid to either the Red Cross or the ministries of health of the involved countries. The Big Four delayed their decision, but finally agreed to provide aid and, against his protestations, Hoover was dragooned into organizing the typhus relief effort. Hoover was unable to locate suitable civilian expertise, so he turned to Gen John Pershing, commander of the American Expeditionary Force (AEF), who identified Col Harry L. Gilchrist as the ideal man for the mission (Appendix A, Figure A4). Gilchrist had experience in public health work while serving with the U.S. Army in Manila and in providing disaster relief after the San Francisco earthquake. Gilchrist had also conducted the U.S. Army’s delousing program in France after the armistice. On 25 June 1919, Wilson endorsed the mission, but there were further delays to obtain the approval of the US Secretary of War, Newton Baker. In late June 1919, Gilchrist received orders to organize a typhus relief mission for Poland, henceforth known as the American–Polish Relief Expedition (APRE). Gilchrist estimated he would need about 500 enlisted men and 25–30 officers to execute the operation. On 16 July, Gilchrist proceeded to Paris to rendezvous with Hoover and Baker approved Gilchrist’s request for personnel, but he was restricted to volunteers. Gilchrist recruited Col Harry Snively of the Army Medical Corps to serve as his deputy, and he sought officers fluent in French, German, or Polish. Most of the officers had served in the Army delousing program and had the appropriate expertise for the project. However, many of the enlisted volunteers lacked the necessary qualifications, and some became discipline problems during the mission [24,38].

By late July 1919, Gilchrist had secured his requisite cadre of volunteers. The US Army donated 3000 beds, 10,000 hair clippers, 500 portable baths, 1.5 million suits of underclothes, and 250 tons of soap to the Polish government [16]. The Poles also purchased surplus supplies from the AEF, including: 27 mobile steam laundries; 500 mules; hundreds of carts; 10,000 beds; 40,000 towels, sheets, blankets, and pillow slips; 100 tons of soap; 17 motorized bathing plants; 300 portable bathing plants; 4000 barrel disinfectors; 50 tons of washing soda; 1 million suits of underclothing; 160 trucks; 320 ambulances; 160 automobiles; and five mobile machine shops [38,57]. To transport this vast quantity of matériel to Poland required 33 trains with a total of 1291 cars. However, due to a paucity of rolling stock, many of the vehicles and supplies traveled to Poland by motorcade, but this was fraught with problems; breakdowns, accidents, and a lack of discipline among the troops all created delays. Gilchrist arrived in Warsaw on 10 August 1919, and met with Polish Minister of Health Tomasz Janiszewski to coordinate the relief effort. Gilchrist then reconnoitered the countryside to observe the living conditions of the Polish people. In Galicia, Gilchrist entered the bleak netherworld inhabited by the Polish peasant: “Homes, which for the most part are only rudely constructed affairs, without furniture of any kind, offer shelter for thousands of these sufferers. They have no beds and lie on straw or hay-strewn floors. Many of these small huts consist of but a single room… The present article of the diet in the majority of cases consists of potatoes, beets, or other vegetables and these in starvation quantities. They seldom see bread. Meat is furnished only occasionally. At present, nearly every house has one to five cases of typhus fever, all lying on the usual beds of hay or straw. Houses have been visited by me in the entire family was stricken, some delirious, and without medical attention of any kind… Doctors and nurses are unknown to these people. The peasants act as combined doctors, nurses, and undertakers. If the dead have no near relatives to claim their effects, the bodies are stripped before burial and their clothes taken away in ignorance by the peasants to distant parts to be sold, thus implanting the disease to new areas” [38]. Due to the impact of the epidemic, whole towns were affected, with schools and businesses shuttered [32]. (For an account of the conditions in a Polish hospital in 1919, see Appendix C, Quote 2).

Gilchrist wanted the APRE to achieve both typhus control and modernization of the Polish healthcare system. For the latter, health agencies were reorganized into a single unit with a central committee to plan their operations and the agencies to implement them. The central committee consisted of Gilchrist and four Polish physicians and was supported by the Departments of Propaganda, Transportation, Statistics, Hospitalization, Quarantine, Finance, and Schooling. This bureaucracy directed the health and sanitation programs throughout Poland [38]. The Polish government appointed typhus commissioners who were given summary judicial powers; their policies pertaining to disease control became law in their districts. The action plan of the typhus commissioners was directed by the Central Committee attached to the Ministry of Public Health [58]. Poland was divided into six districts of 18 to 25 counties each, with a chief medical officer designated for every county. American Army physicians provided expertise on typhus control and other medical issues to the county health officers. The Polish government purchased a fleet of vehicles and mobile bathing facilities in an initiative to motorize its health service. However, this proved more difficult than anticipated. Many of the vehicles had been damaged en route to Poland, and there were shortages of spare parts, mechanics, and trained drivers [38].

In summer 1919, the Polish government enacted several public health policies, including mandatory smallpox vaccination and a law requiring the reporting of infectious diseases cases and deaths. On 1 August 1919, the Polish Ministry of Health created the Central Anti-Typhus Committee (Cekadur) [11]. On 24 September 1919, the Ministry of Health issued regulations for combating infectious diseases (however, there were no specific penalties for non-compliance):

Article 1—All inhabitants of Poland are obligated to thoroughly clean themselves, their apartments, appliances, clothing and underwear of dirt and vermin.

Article 2—All proprietors, administrators and residents of cities, residents of settlements and villages are obligated to clean the dirt from their homes, farm buildings, courtyards, toilets, gutters, sewers, dumps and all streets, spaces and reservoirs containing contamination [16].

Patients, their families, physicians, nurses, teachers, and religious leaders were obliged to report typhus cases to the head of the community. However, Poles were often reluctant to report because if the local medical officers acted according to protocol, the infected person had to submit to hospital isolation, exclusion from work or school, the marking of their homes, and the disinfection of their possessions [41].

In October 1919, Gilchrist’s unit consisted of 500 enlisted men and 32 officers, including nine physicians [35]. The first step of typhus control was the establishment of a cordon sanitaire along Poland’s eastern border, which included barracks, hospitals, and delousing stations along the principal railway lines [15,24]. In 1919, the APRE set up 15 delousing stations [35]. Initially, coal was in short supply, so it proved difficult to induce the populace to bathe with cold water. Several delousing and bathing trains were constructed, which utilized steam from the locomotive; each was capable of delousing 400 persons per day [24]. To accomplish his mission, Gilchrist stated that he needed soap in the thousands of tons, hospital linens and underwear in the hundreds of thousands of sets, and equipment for thousands of hospital beds [59].

Passenger train cars were sanitized, and the passengers received medical inspection. Sanitizing of trains and their depots was achieved with carbolic acid, cresol, lime, and sulfur combustion [24]. Rail cars were also disinfested by placing them into blocked-off tunnels and then introducing hydrogen cyanide gas [60]. Dwellings were also fumigated with hydrogen cyanide, which necessitated sealing up the buildings using a paste of clay and horse manure [24]. Fur clothing was deloused by treating it with gasoline or naphthalene and placing it in a sealed chest [24,60].

**Figure 4:**
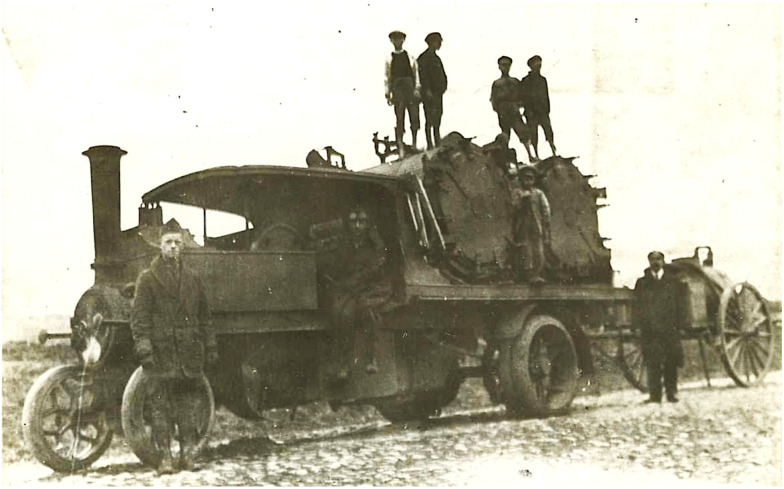
One of the 10 mobile Foden–Thresh Mobile Sterilizers sent to Poland by the APRE in 1919. The Foden lorry was steam powered and supplied steam to the Thresh disinfector [24,61].

The APRE organized four mobile field units in its battle against pediculosis. Each unit consisted of one commissioned officer, three noncommissioned officers, and 15 enlisted men and was equipped with a motorized bath plant, tents, stoves, bedding, and disinfectors [38] (Figure 4). The commander preceded the unit to each town and conferred with the local medical officer and town leaders. A site was selected for the bathing facilities, and then the commander and the town officials had the difficult task of convincing the residents to submit to delousing due to their natural fear of the unknown [38]. Furthermore, bathing was often associated with the coercive measures employed during the German occupation. In one town of 7000 inhabitants, physicians reported that it was likely none of the adults had bathed in more than a year [35]. The reticence of the local populace to cooperate was quite frustrating to the Americans, so the bread ration was withheld from those who did not present a certificate from the bathing units [38]. When persons with typhus were diagnosed, their houses and possessions were disinfested. The APRE teams also treated other common illnesses, such as trachoma, conjunctivitis, otitis, tuberculosis, skin disorders, goiter, and anemia. However, there were many impediments to the work of the mobile field units. Vehicles frequently broke down on the rough roads, and gasoline was in short supply. In some areas, Catholics and Jews demanded segregated treatment. Laws requiring compulsory bathing were often ignored because there was insufficient fuel to heat the water. The disinfestation work sometimes had to be abandoned because of severe weather, an absence of shelters for those being bathed, or a lack of cooperation from local officials. There was often suspicion by the villagers about the intent behind the disinfections; when teachings about hygiene were unsuccessful, sometimes offerings of food or soap were a sufficient inducement [35]. Working with refugees proved to be even more challenging; Gilchrist estimated that by January of 1920 a half-million refugees had entered Poland without medical inspection [58]. Weakened by exposure, malnutrition, and disease, the refugees “died by the thousands, their bodies being piled in great heaps in adjoining buildings, awaiting burial” [35].

In early 1920, Gilchrist created a mobile unit based in Wilno (present-day Vilnius, Lithuania) that operated four railcar-mounted delousing plants. The Polish government ordered that all trains transiting Wilno be sanitized, so three APRE staff were assigned to run a sterilizing station there, treating about 200 railroad cars a week. By the time the sanitary trains were withdrawn from Wilno in the early summer of 1920, 32,000 persons had been deloused. Trains also deployed delousing equipment to other cities where the rail lines entered Poland. Poles were trained to operate delousing facilities in order to release APRE personnel for other assignments [35].

The delousing process began with hair clipping, although Jews were allowed to retain their locks for religious reasons. The shorn and unshorn then entered tents, where American attendants (or Poles, in the case of women) directed them through quick showers and disinfested their clothing. After bathing, the people dressed, but were allowed to keep their bar of soap, a precious item at that time [62]. The delousing units were able to process 500–800 persons each day. In early November 1919, unexpected winter storms forced the Americans to discontinue field operations [38].

**Figure A5:**
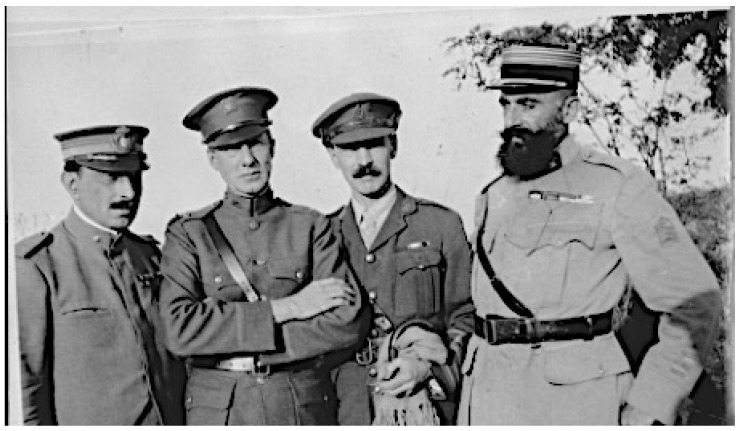
Members of the Interallied Medical Commission sent by the League of Red Cross Societies to Poland. Left to right: Lt. Col. Aldo Castellani; Col. Hugh S. Cumming; Lt. Col. George S. Buchanan; Col. Fernand Visbecq. Summer 1919 [144].

Meanwhile, in June 1919, the LRCS sent the Inter-Allied Medical Commission (IAMC) to Poland to survey the typhus situation. The IAMC consisted of Drs. Hugh Cumming (Chairman and Asst Surgeon General, US Public Health Service), George Buchanan (senior medical officer, British Ministry of Health), Aldo Castellani (Professor, London School of Tropical Medicine), and Col Fernand Visbecq (Medecin Principal of Service de Santé, French Army) (Appendix A, Figure A5). Rajchman accompanied the Commission on its month-long inspection of Poland [12,42]. The conditions were so wretched that the IAMC telegraphed an urgent interim report to LRCS headquarters on 9 September confirming that typhus was widespread and advising action by the LoN to coordinate Red Cross efforts in Poland and to maintain delousing stations for refugees and the repatriated. The IAMC also requested 2000 tons of soap; shirts, drawers, outer clothing, and blankets (200,000 of each); equipment and utensils for 100 hospitals of 300 beds each; salvarsan (to treat relapsing fever and syphilis); iodides (to treat delirium); quinine (for malaria), castor oil (for constipation), salol (an analgesic), opium; and washtubs for boiling clothes [63].

**Figure 5:**
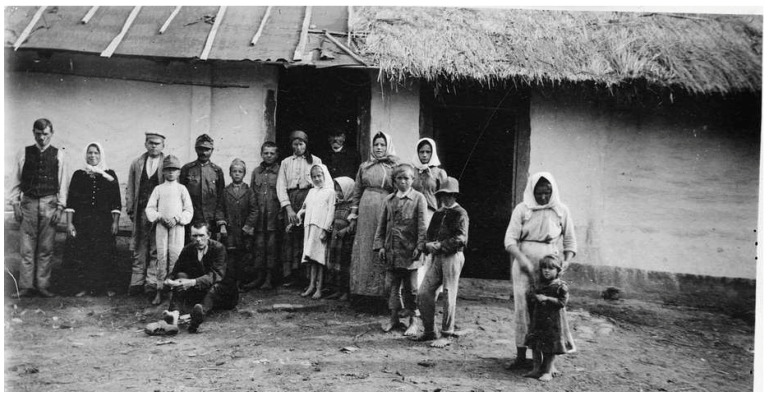
Original caption: “The Interallied Medical Commission… found that overcrowding is a large contributing factor to the spread of the epidemic. Most of the houses in Suchodoly in Eastern Galicia were destroyed by artillery fire and all the people in the above photograph, comprising three families, are living in this two-room hut” [64]. Summer 1919.

**Figure 6:**
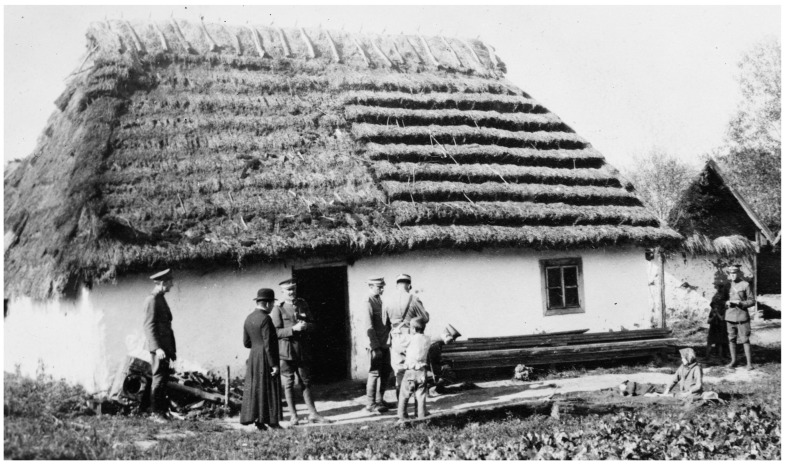
Original caption: “A typical instance of the scourge of typhus found by the Interallied Medical Commission… The little boy seen… lying on the ground is in the acute stage of typhus, his sister sitting beside him is convalescing, his mother inside the cottage is very sick with typhus, and his father died of typhus and was buried the same day on which this photograph was taken” [65]. Poland, Summer 1919.

The findings of the IAMC were published in October 1919 with the entire issue of the Bulletin of the LRCS devoted to the Report of Medical Commission to Poland. The account described the swirling maelstrom of disease, overcrowded housing and trains, malnutrition, near universal lousiness, filth, poverty, and the ongoing refugee crisis that had engulfed Poland (Figure 5 and Figure 6). The Commission also found that relapsing fever was even more common in some areas than typhus and that the treatment for relapsing fever, salvarsan, was not readily available. The IAMC had visited Poland in late summer, when typhus cases are usually low. Thus, the report concluded that the situation was likely to worsen before improving. The IAMC recommended: strengthening the sanitary cordon; increasing the availability of clean clothing for refugees; establishing a safe water supply; constructing drainage systems; establishing public baths, clothing wash houses, and contagion hospitals; implementing large-scale vaccination against smallpox and enteric diseases (cholera, typhoid, and dysentery); and providing food aid. The Commission also recommended that the LRCS recruit 50 physicians and nurses to undertake infectious diseases work in Poland for a year [63].

In November 1919 LRCS Director David Henderson stated that the crisis of epidemic disease in Europe was beyond the resources of the LRCS, and that governmental action was needed without delay [49]. The LRCS Medical Department was headed by Dr. Richard Strong, who had directed the International Sanitary Commission in Serbia during its 1915 typhus epidemic and the ARC Trench Fever Commission, so he was well-versed in the control of louse-borne diseases. Strong organized both relief and scientific missions to Poland. The relief operation had the following components: disinfestation of persons in highly affected districts; establishing and operating contagion hospitals; the removal of typhus patients to these hospitals; disinfestations of patient contacts; and the establishment of delousing and quarantine stations strategically located near refugee routes. In conjunction, there was a multimedia educational campaign with pamphlets and circulars, demonstrations, and films [32], but this was challenging in a nation in which half of the population was illiterate [40]. To encourage compliance for delousing, a play was performed depicting the menace of typhus and the louse and the measures needed to combat the epidemic. The British Red Cross Society donated £100,000 ($8 million) for the establishment of hospitals, and the British government donated an additional £20,000 ($1.6 million) and extended £100,000 ($8 million) credit to purchase medicaments and supplies. The LRCS shipped 378 train cars of supplies to Poland [32]. However, Poland remained woefully short of doctors, with only one per 150,000 inhabitants; the baseline physican shortage had been further exacerbated by physicians succumbing to typhus. In Galicia alone, 46 physicians had perished from typhus by early 1920 [66].

Dr. Strong appealed to infectologists, field sanitarians, nurses with fever hospital experience, and sanitary engineers knowledgeable about water purification to join the mission. The Polish government requested teams instead of individual specialists. Red Cross societies from the USA, Australia, Belgium, Britain, Italy, Japan, Portugal, Rumania, Serbia, Sweden, and Spain all furnished support [32,67]. The LRCS sent a commission to Poland from January to October of 1920 to advise the Polish government, composed of US Army Medical Corps personnel: Col Henry Shaw, Lt Col George Fordham, Maj R.M. Taylor, and Maj L.H. Dunn. However, sanitary measures proposed by foreigners were often not accepted by the Poles. Shaw reported that there was almost universal antagonism with regard to bathing and delousing due to prior negative experiences with enforced bathing by Russian and German authorities [68]. Due to their ignorance of the principles of hygiene and suspicion of foreigners, many Poles resorted to superstition and religion for their salvation from typhus. Efforts by the LRCS to recruit priests and rabbis into the anti-typhus campaign failed. Orthodox Jews mistrusted the contagion hospitals, and there were concerns that Jews were concealing persons with typhus and forging delousing certificates [7].

## 4. The Typhus Research Commission of the League of Red Cross Societies

**Figure A6:**
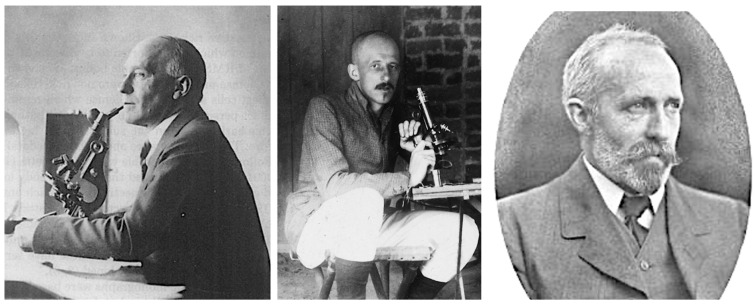
Members of the Typhus Research Commission of the League of Red Cross Society. Left to right: Pathologist S. Burt Wolbach, Harvard University [145]; Pathologist John L. Todd, McGill University [146]; entomologist Arthur Bacot, Lister Institute of Preventive Medicine [147].

For the scientific arm of the LRCS endeavor, called the Typhus Research Commission, Strong recruited Harvard University pathologist S. Burt Wolbach and McGill University parasitologist John L. Todd to undertake studies of the etiology and pathologic effects of typhus [69]. Wolbach was selected because he had made the seminal discovery in 1916 that the rickettsiae of Rocky Mountain Spotted Fever caused vasculitis [70,71]. Todd and Wolbach had previous fruitful collaborations, including an expedition to Mexico in which they found organisms consistent with rickettsiae in cutaneous vascular lesions in persons with typhus [72]. In January 1920, Drs. Wolbach, Todd, and Francis W. Palfrey (an instructor at Harvard Medical School), along with three assistants, sailed to Europe. In Paris, they were joined by Arthur Bacot, entomologist of London’s Lister Institute of Preventive Medicine (Appendix A, Figure A6).

From Paris, they proceeded to Wilno; there they found that the hospital in which they had planned to work had been ransacked by the retreating Germans. The investigators dolefully returned to Warsaw, and there they were provided laboratory space in the new public health building by Rajchman. It was also arranged for the team to control one wing of the St. Stanislaus Hospital, the principal fever hospital of Warsaw, at that time entirely devoted to typhus care, with a capacity of 300 beds. From the ARC mission in Poland under Col Albert Chesley, they obtained nurses, supplies, and access to vehicles. The investigators were granted full autopsy privileges for all fatal cases. The decision to assume control of a dedicated ward of the hospital instead of working in the regular Polish hospital wards was reached for the safety of researchers and their nurses, because, in general, Polish hospitals did not perform sufficient delousing. The appropriate protocols were in place, but the hospital orderlies often neglected to rigorously carry them out. Although typhus occurred amongst the medical personnel in the general wards of St. Stanislaus Hospital, there were no cases among the LRCS research personnel during their time in Poland [70]. The investigators arrived in February 1920, near the peak of the epidemic, with 35,000 new cases of typhus reported in Poland in the month prior [36,73].

**Figure 7:**
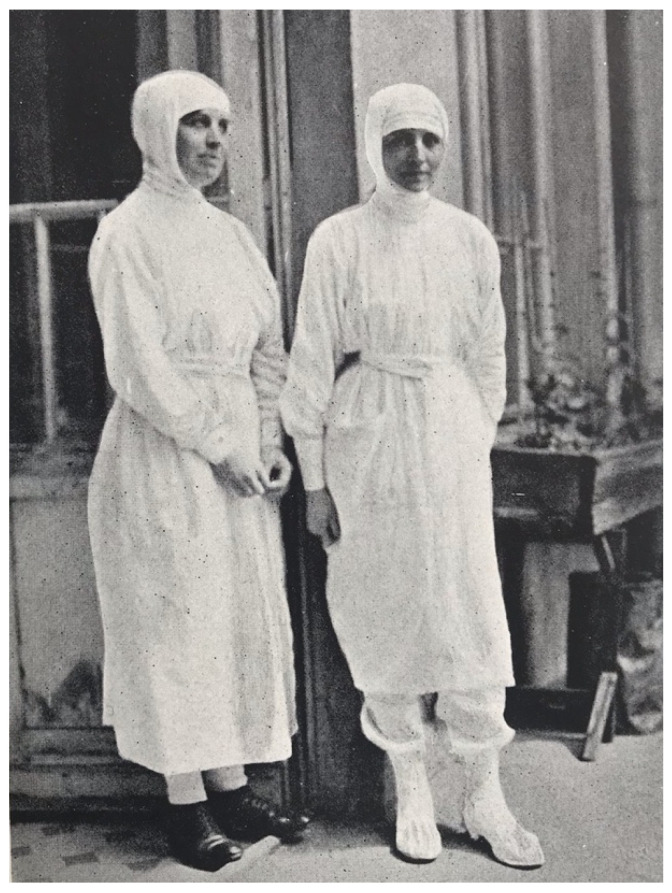
Louse-protective attire worn in the St. Stanislaus Hospital, Warsaw, Poland, 1919 [69].

The delousing protocol performed by the LRCS team at St. Stanislaus Hospital was quite laborious. In the delousing room, the patient was stripped of clothing before being transferred to a table. The clothing was conveyed to a steam sterilizer. On the table, all hair thick enough to conceal lice or their eggs was clipped to below the egg-bearing length, and the clippings were either boiled or burned. The body, except the scalp, was bathed with soap and water. Soap and water do not kill lice; they only facilitate mechanical removal, and if the clipping had not been meticulously conducted, viable lice and their eggs may remain amongst the hairs. After the bathing and drying, the scalp, axillae, and pubic and anal regions were doused with kerosene or a light oil, and the same was rubbed lightly over the whole body. The patient was then transferred to a clean stretcher, given a head-cap and night shirt, and taken to bed. The table and floor of the delousing room were cleaned with kerosene cloths and mopped after each patient. The delousing of each patient was performed by two nurses or orderlies; ideally, these were persons who are typhus-immune by virtue of prior infection, and they wore louse-proof gowns (sterilized by steam or naphthalene after each use) and rubber gloves (Figure 7) [69,70].

The research commission brought uninfected lice, and these lice were fed on members of the commission in order to maintain an infection-free colony for experimental purposes [69]. Bacot brought a colony of pathogen-free lice that he had fed upon himself for over 4 years, but his participation was cut short when he contracted trench fever, acquired in the course of collecting lice from underwear in a public bath in Warsaw. During the attack of trench fever, Bacot fed uninfected lice upon himself and definitively showed the appearance of a rickettsia-like organism in the lice, proving the etiology of trench fever [73]. Meanwhile, Wolbach and his colleagues studied a cohort of 181 patients with typhus and quantitatively recorded the signs and symptoms of typhus [69]. Wolbach was also able to perform 38 complete autopsies within a few hours of their deaths [70].

Wolbach confirmed the histologic findings made by Henri da Rocha Lima of *R. prowasekii* in the louse gut [74] and found organisms with the same characteristics in vascular lesions of humans with typhus. Wolbach thus concluded that *R. prowasekii* is the cause of typhus and determined that typhus, like RMSF, induced a diffuse vasculitis in multiple organs (skeletal muscle, myocardium, brain, kidneys, adrenals, thyroid, testes, and ovaries), and this explained how the infection caused such severe morbidity and mortality [69]. Palfrey reported that the most frequent causes of death were bronchopneumonia and “passage into a state of stupor in spite of the disappearance of fever”. When the complete clinical, experimental, and histopathologic findings of the Research Commission were published in 1922, it proved to be a tour de force of infectology [69,75]. The importance of the Typhus Research Commission to establishing the etiologies of both typhus and trench fever was acknowledged in an editorial in the Journal of the American Medical Association in 1922 [76].

## 5. The Conclusions of American–Polish Relief Expedition Activities

Meanwhile, winter storms in 1919–1920 curtailed the operations of the APRE mobile field units shortly before their scheduled departure. However, the Polish government requested continued American assistance, so the Polish Minister of Health convinced US Secretary of War Baker to extend the term of the APRE to 1 November 1920. Nevertheless, the complement of the APRE was reduced to 161 men in November 1919 and further in December. Despite the smaller staff, Gilchrist continued all of the health-promotion activities except for the mobile field units. APRE personnel continued to inspect health facilities, provide advice to local authorities on sanitary matters, and instruct the Poles on delousing methods [24].

**Figure 8:**
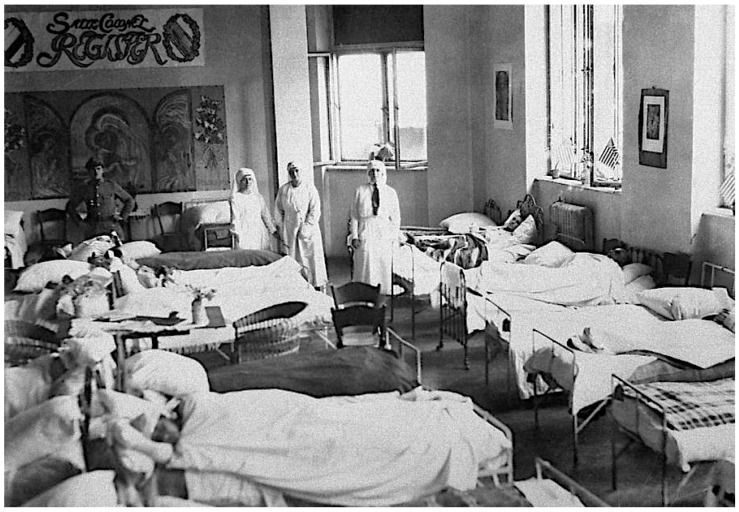
Grace Hospital in Detroit provided funds for a 100-bed hospital for 6 months. A banner honoring Col. Register hangs on the wall. The original location was Tarnapol, but after the Bolsheviks seized this city, it was moved to an auditorium in Warsaw [78]. Early 1920.

The focus of American and Polish health workers during the winter of 1919–1920 was the maintenance of the Eastern cordon sanitaire. To achieve this goal, Gilchrist assigned Lt Arthur Fox and 18 enlisted men to a mobile unit that included four railcar-mounted delousing plants, which were located in cities where rail lines entered Poland. In January 1920, APRE member Lt Col Edward C. Register died of typhus [38]. Register had been stationed at Tarnopol, where typhus had been raging for months. There he was charged with organizing a 1500-bed hospital, but 15 days after he arrived in the city, he contracted the infection [77] (Figure 8). Out of 12 other doctors in Tarnopol, 10 succumbed to typhus [58].

When the Polish Prime Minister, Ignacy Paderewski, attended the first session of the LoN in January 1920, he brought Rajchman to present the gravitas of the situation in Poland. At that time, Poland was spending 1.5% of its national budget on typhus control, but the epidemic was inexorable. In January 1920 alone, there were 100,000 cases of typhus in Poland, with 12,000 deaths [42]. Despite Rajchman’s pleas, the LoN took no action. Subsequently, the LoN convened two international health conferences in which the LoN Council committed to an international campaign against typhus in Poland. The resolution of the Paris Conference in March 1920 stated that prevention of the spread of typhus from Poland was urgently necessary. The Council deemed that the LoN was the organization most qualified to undertake this work. At a meeting in Rome 1 month later, the concept of an Epidemic Commission was formulated to execute the plan [19].

In March 1920, Henry Davison of the LRCS conceded that taming the epidemic situation in Europe was well beyond the resources of the LRCS, and he called on the LoN and all nations to take up the fight against pestilence and hunger [79]. Private aid continued to trickle in. In March 1920, the Joint Distribution Committee of the American Funds for Jewish War Sufferers provided a $100,000 ($1.6 million) grant to the APRE [80], which Gilchrist used to purchase coal for the delousing facilities [81].

An ARC Commission had been established to aid Poland in March of 1919. The ARC set up five canteen trains that were feeding thousands daily. By 30 June 1920, ARC workers contributed to the founding of 268 hospitals with 26,128 beds and operated three teaching hospitals with 1076 beds. They also assisted 30 dispensaries and set up 82 new dispensaries. Supplies were furnished to 22 sanitary and bath trains. Relief reached 2810 towns, where nearly 700,000 persons were provided with clothing and other provisions [82].

At the International Health Conference convened in London on 14 April 1920, Rajchman and the Polish Vice Minister of Health Witold Chodzko presented the dismal conditions prevailing in Poland. The meeting included representatives from the USA, Great Britain, Japan, Italy, France, and the LRCS [12,36]. The conferees called on the LoN to take action, calling the control of typhus in Poland a matter of global concern [36]. To proponents of the LoN, the epidemic of Poland represented an opportunity to demonstrate that the new institution could implement a coordinated government-sponsored international response that was more effective than the prior practice of sporadic charitable relief. The interventions suggested by the conference delegates included hospital construction, disinfection, and quarantine through the allocation of £3.25 million ($187 million) for materials and personnel. However, after the conference, the proposed action of the LoN on Poland’s behalf remained controversial. Edwin Embree, Secretary of the Rockefeller Foundation, argued that, because typhus was previously endemic to Poland, the current epidemic was no more deserving of international action than measles in England [14].

On 25 April 1920, Poland endeavored to exploit the chaos transpiring during the Russian Civil War to acquire territory along its eastern border and invaded Soviet Ukraine. After initial successes, the Polish army was repulsed and during the early summer of 1920, Bolshevik forces advanced to the gates of Warsaw [83]. The bathing trains were captured, and many of the delousing facilities and quarantine stations of the APRE fell into Russian hands [38]. Before the Bolshevik invasion, the Poles had equipped 92 infection hospitals with 5870 beds; most of these were also lost in the Russian onslaught [84]. It was estimated that three-quarters of the infrastructure of the cordon sanitaire was destroyed [42]. Historian Christopher Blackburn has suggested that the Soviets specifically targeted anti-typhus facilities and executed Polish physicians in order to conduct a crude sort of biological warfare against the Poles [85]. The war resulted in louse-ridden troops mobilizing throughout the country, negating the prior control measures [35]. Furthermore, during the Polish–Soviet War, 200,000 refugees entered Poland without medical inspection [86]. The war also created an internal refugee crisis that uprooted eight million Poles and Ukranians [46]. With the ongoing war, Col. Henry Shaw of the LRCS was skeptical that an effective anti-epidemic campaign could be mounted until hostilities ceased because of the destruction and capture of sanitary infrastructure, the disruption of sanitary activities, and the lack of fuel to operate delousing centers [14]. Thus, the LRCS suspended its anti-epidemic activities in Poland [87].

As a result of the Polish–Soviet War, by May 1920, the American sanitary cordon centered at Wilno was completely disrupted. In a report to the LoN, Gilchrist warned that unless the typhus epidemic was checked in Poland, it would threaten the whole of Europe. The Polish epidemic, now in its fourth year, was intensifying each year. Gilchrist blamed the westward march of typhus on the unstable political situation in the Ukraine. He appealed for further aid to Poland, asserting that it was a rampart against a disease which threatens the world [19,24].

However, by the summer of 1920, the momentum of the APRE crusade was flagging. The ongoing Polish–Soviet War, waning enthusiasm for American entanglement in European affairs, and a conflict between the short-term goal of epidemic control and the long-range objective of improving overall public health, were all interfering with the campaign [24,35]. Without its duties along the frontier because of the war, the APRE initiated projects within the interior of Poland. It conducted delousing of a refugee camp near Krakow and disinfested: members of Haller’s army (Polish troops that had served in France); Polish conscripts of the Austro–Hungarian Army that had become Allied prisoners-of-war; and Polish–American volunteers who were engaged in fighting the Bolsheviki. In August 1920, Gilchrist established a delousing program for the Polish Army. By October 1920 virtually all activities of the APRE had ceased. On 2 November 1920, all of the Americans except Gilchrist and four enlisted men had left Poland; the latter stayed to complete administrative matters and to dispose of the remaining supplies. On 4 January 1921, the APRE was disbanded [35,38].

Despite all the labor that had been expended, the work of the APRE was tarnished by the misdeeds of some of its personnel: thievery, drunk and disorderly conduct, cavorting with the local women, and violence against Polish civilians [24,38]. A US Inspector General’s report was highly critical, concluding that the APRE had failed to accomplish its goals. The report offered several reasons for the failure: it was planned in France without knowing the actual situation in Poland; many of the personnel were unsuited to the task; the unreliability of the motor vehicles; and conducting activities through the Polish Ministry of Health instead of the Polish Army, which had greater authority. It was true that Gilchrist was unable to maintain the discipline of his units, but he was spread too thin. In addition to his military command, he was also the liaison to the Polish Ministry of Health and the director of a delousing campaign over a large geographic area in a nation racked by poverty, hunger, and war. The unforeseen Polish–Soviet War also proved disastrous to the operation. Nevertheless, although the APRE was not a total success, it clearly contributed to reversing the typhus epidemic in Poland under very difficult circumstances. It deloused thousands of refugees along the eastern border and civilians in the hinterlands, and it also helped to modernize and motorize the Polish health system [24,38]. An editorial in the Lancet called the APRE: “A most creditable record of industry, good organisation, and philanthropy” [88].

## 6. The Creation of the League of Nations Epidemic Commission

In June 1920, the LoN created the Epidemic Commission (EC). Two Britishers of military background, retired Royal Army Lt-Col Kenyon Vaughan-Morgan and physician F. Norman White (former Sanitary Commissioner of India), assumed the positions of chief and medical commissioner, respectively. Rajchman was later appointed as a second medical commissioner. Measures recommended by the EC included: a chain of quarantine stations; fixed and mobile hospitals; facilities for disinfestation; and provision for coordinating the work of the governmental and relief organizations [36]. The EC estimated that £3.25 million ($187 million) was needed to complete the operation. Items requested by the EC included: 272 vapor and 72 hot air disinfection apparatuses; 500 mobile disinfection units; 100 showers with 12-person capacity; 30,000 hospital beds; 6000 tons of staple foods; two million shirts; 1.5 million sets of underwear, and over 100 ambulances and other vehicles [42]. After the conference, Dr. White returned to Poland with Rajchman and surveyed the worst-affected areas of eastern Poland in May through June of 1920. White was further convinced that urgent international intervention was needed to stem the tide of infection in Poland before it penetrated deeper into Europe [42]; he presented the dire situation in Poland to the general public at a lecture at the University of London in July 1920 [59], but there was no resulting outpouring of aid. Poland continued to mobilize its own populace for its public health crusade; with LRCS support, the Polish Red Cross grew from 30,000 members in March 1920 to 900,000 by July [31,88]. By October 1920, the Polish Red Cross was operating 20 hospitals with 3025 beds, three sanitary trains, four bathhouses and laundries, multiple tuberculosis sanitoria, and several clothing workshops [89].

To raise the requisite £3.25 million ($188 million) to combat the epidemic, the LoN requested a contribution proportionate to a nation’s risk of contagion and ability to pay. Thus, Germany was expected to pay £579,000 ($33.3 million); Britain £260,000 ($15 million); France £152,000 ($8.7 million); Poland £121,900 ($7 million); USA £114,350 ($6.6 million), among others [38]. However, the funds were slow in coming. In August 1920, Denmark contributed £5000 ($287,000), but Australia, New Zealand, and Japan declined to donate because of their distance from the epidemic. Italy balked because of its own domestic economic woes [14]. Several countries sought consideration for prior donations. Britain had previously supplied 3.2 million yards of fabric, 200,000 pairs of shoes, and £500,000 ($28.7 million) for transportation infrastructure to Poland and Rumania. The USA had already funded the ARA and the APRE. The French had contributed medical supplies worth 1.3 million francs ($1.8 million) [42].

In June 1920 Britain was ready to release £50,000 ($2.9 million) for the cause, and Lord Balfour called on Léon Bourgeoise, President of the French Senate, to make a comparable donation. The French debated the issue, but an impasse occurred when Britain and France pledged to contribute £50,000 ($2.9 million) only if three other countries agreed to do likewise. In July 1920, Vaughan-Morgan resigned from the EC due to frustration over funding; he was replaced by F. Norman White. France demanded a position on the EC and recognition for prior donations; French physician Col Aime Gauthier was then appointed medical commissioner [42]. In September 1920, George Newman of the British Ministry of Health requested that the Rockefeller Foundation send funds to Poland but they declined, citing Poland’s unstable military situation and the non-participation of the USA in the LoN [7].

Meanwhile, in 1919 and in 1920, the ARA facilitated Polish economic recovery by negotiating several loans and trade agreements between Poland and other European countries. The USA also donated 2000 tons of cotton, 500,000 tons of flour, 300,000 tons of wheat and rye, and 3000 tons of lard. A program was also instituted to sell Polish bonds in the United States. From the end of World War I to the conclusion of the Polish–Soviet War, the USA delivered over 750,000 metric tons of supplies to Poland, valued at $200 million ($3.1 billion) [16]. All of these measures served to revitalize the Polish economy, providing much-needed funds for the government to directly fight the epidemic and improve housing and nutrition programs.

**Figure A7:**
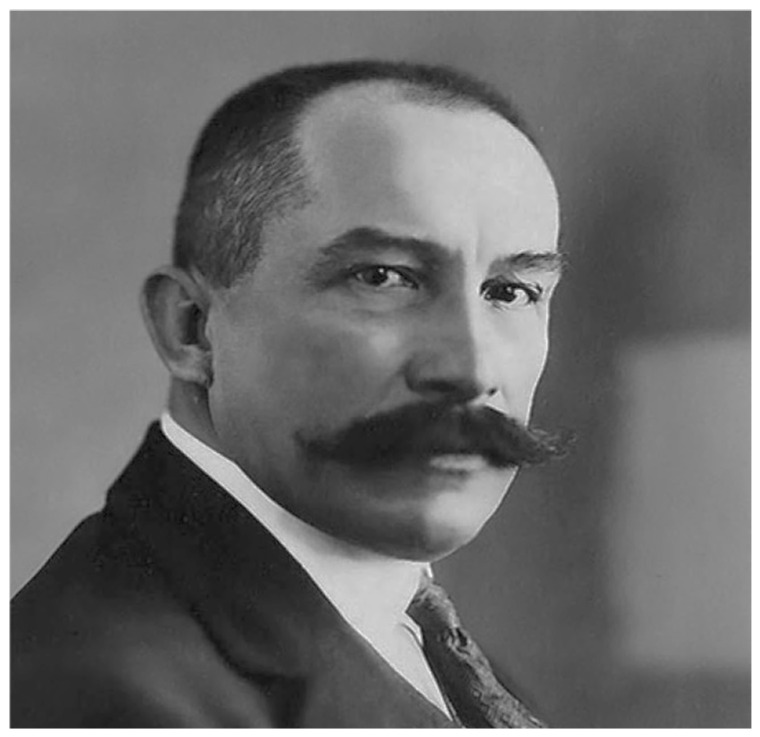
Polish physician Emil Godlewski, Jr, appointed the Extraordinary Commissioner to Fight the Epidemics by the Polish government in 1920 [148].

By mid-1920, it was evident that the Polish Central Anti-Typhus Committee (Cekadur) was not achieving the desired results. The Cekadur’s committee system was unable to effectively attack the epidemic; collegial discussion of each issue, incorporating different views of the involved ministries, and agreeing on the powers of various authorities, led to ponderously slow interventions. In place of the Cekadur, in July 1920, the Polish government created the office of the Supreme Extraordinary Commissioner for the Fight Against Epidemics. The Commissioner was given broad powers: mobilizing the production of materials necessary to combat infectious diseases; the authority to issue regulations to combat epidemics; appointing commissioners for specific areas of the country; appointing medical and technical advisors; and the use of requisite military, medical, and auxiliary forces [90]. The Commissariat was headed by Prof. Emil Godlewski Jr, who was soon designated “the typhus tsar” [91] (Appendix A, Figure A7). Godlewski had practiced as a physician in a military hospital in Krakow and was also director of the Sanitary Section of the Prince-Episcopal Committee of Help during World War I and its immediate aftermath. In this capacity, he had established mobile sanitary units and infection hospitals, so he had direct experience in typhus control [92,93]. Furthermore, in the most severely affected regions of Poland, competent regional commissars were now appointed to organize local control activities. By the end of 1920, the Commissariat was operating 188 hospitals with 9245 beds, with additional beds in Red Cross and military hospitals, for a total of 12,915 beds [94].

## 7. A Fateful Year, 1920, Draws to a Close

**Figure A8:**
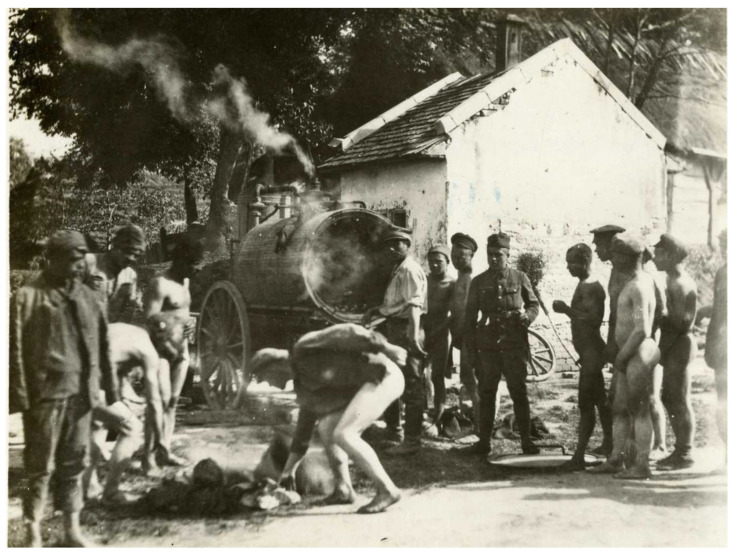
American–Polish Relief Expedition team delousing Bolsheviks along a road in Poland, 1919–1920 [61]. A Thresh steam disinfection apparatus for clothing is seen in the background.

In August 1920, the Polish Army had achieved a resounding victory over the Russians in the Battle of Warsaw and drove them out of Poland; a ceasefire was enacted in October 1920. Although the Treaty of Riga would not be signed until March 1921, the cessation of hostilities was crucial for the improvement of public health in Poland, because the Polish government no longer needed to marshal its resources for its military survival. Poland suffered 250,000 casualties in the war, with 48,000 deaths. The Polish–Soviet War had consumed 62% of Poland’s national budget from mid-1919 to March 1920 and damaged Poland’s standing to receive aid from abroad [95]. By the end of the war, about 80,000 Bolshevik soldiers had been captured [96]. The APRE deloused some of these Bolshevik prisoners (Appendix A, Figure A8) [61].

By 1920, the Polish health authorities had fielded 294 mobile field sanitary units, which had deloused 311,374 persons and 72,700 dwellings; another 19,000 and 15,860 were disinfested by the APRE and the British Quakers, respectively. Railcars were being sanitized weekly [32]. The year 1920 also proved to be decisive in the Russian Civil War, ending in Bolshevik domination over multiple enemies. Although in the long term the conclusion of the war was crucial in the ultimate resolution of the typhus epidemic on the continent, in the short term, the defeat of anti-Bolshevik forces created yet another refugee crisis, which had significant consequences for the Polish anti-typhus campaign. Twenty thousand refugees entered Poland after the defeat of White Russian General Denikin’s forces in March 1920; 8000 required hospitalization, 6000 of them for typhus and relapsing fever [84]. The army of the Ukrainian People’s Republic under Symon Petliura had been severely weakened by an outbreak of typhus within its ranks in the autumn of 1919 [97] and was defeated by the Bolsheviks in mid-1920; tens of thousands of Ukrainian refugees then streamed across the Polish border, overwhelming quarantine stations [57].

In late April of 1920, the shattered army of Lavr Bredoff (governor of Ukraine) staggered westward from Kiev, seeking refuge in the Dniester Valley of Poland, trailed by a typhus-ridden retinue of peasants and ex-dignitaries [98]; 20,000 of these refugees entered Poland in 1920 [99]. Gilchrist also reported that the Bolsheviks shipped typhus-infected persons by train to the frontier with Poland, and, cast off by their own nation, many drifted across the border [57]. There was also an infectious diseases crisis within the Polish internment camps holding Ukrainians, Belorussians, Cossacks, and anti-Soviet Russian troops. In March of 1920, in camps holding 19,510 men, there were 1141 documented cases of typhus, relapsing fever, dysentery, and typhoid [99]. In late 1920, in response to a request by the Polish Red Cross to assess the conditions after the Polish–Soviet War, the ICRC sent a fact-finding delegation to Poland, led by Lucien Brunel. Brunel visited these internment camps and described the pitiful conditions of disease and malnutrition therein [97]. (There is a 15-min film that shows various aspects of the ICRC’s relief work in Poland [100]. Highlights of the film are presented in Appendix D). One month after the signing of the Polish–Soviet ceasefire, in November 1920, the Army of the Ukrainian National Republic retreated into Poland, where its soldiers were interned [101]. To decrease the risk of large intra-camp outbreaks of typhus, Polish authorities opened 10 smaller internment camps in 1921 [97].

In 1920, Arthur Balfour, Chairman of the LoN Council, again implored member governments to provide succor to Poland. He maintained that the world, having recreated Poland at the end of World War I, had a responsibility to deliver it to a state of normalcy. Second, he stated that if typhus remained unchecked in Poland it would metastasize to other parts of Europe. Thirdly, he appealed to the spirit of international humanitarianism. Balfour reiterated that the magnitude of this calamity was much beyond the resources of private organizations. He further contended that the strategy to overcome typhus was already in place, but monies were needed to complete the mission [102]. Despite these pleas, by mid-October of 1920, only a few LoN member states responded. Eric Drummond, Secretary General of the LoN, remarked that the campaign against typhus was threatened with failure unless new measures were quickly adopted [42]. Unfortunately, the appeals to aid Poland came at a time when several European countries were donating substantial funds to the cause of prisoner-of-war repatriation. About 1.5 million Austrian, German, and Eastern European prisoners were languishing in transit camps within Russia, which itself was in a state of economic collapse and could not properly care for these unfortunates. Unless repatriation was expedited, tens of thousands were unlikely to survive the upcoming winter [103].

By October 1920, the ARC had established a typhus hospital and sent 76 doctors and nurses to Poland. The British Red Cross, with matching funds from the British government, opened two hospitals. The Red Cross Societies of Australia, Spain, Netherlands, Japan, Portugal, and Belgium all contributed supplies or funds [87].

To convince member states of the urgent need to assist Poland, in November 1920, the LoN sent a commission consisting of Dr. Henri Pottevin (Director, Office International d’Hygíene Publique (OIHP)), Thomas Madsen (Director, State Serum Institute, Copenhagen), and Dr. Norman White (director of the EC), to investigate conditions in Poland [104]. In the northeast, there was great overcrowding due to destruction of housing from years of warfare, including the recent war with Soviet Russia. There was no stored food for the upcoming winter, and the number of horses and cattle were one-quarter of pre-World War I levels. The commission was unable to get accurate statistics on typhus in northeastern Poland, but it appeared to be widespread. In one hospital in Grodno, there were 220 patients with typhus or relapsing fever. In the villages, they found typhus cases in large numbers in almost every house that was visited. In Galicia, the social and economic conditions were similar to northeastern Poland; typhus was geographically widespread, but the recorded density of cases was low. However, the statistics on the disease were probably inaccurate. For example, in one village, which reported no typhus, a house-to-house inquiry revealed multiple cases. They also found that the eastern districts of Poland were awash with cases of typhoid and cholera, necessitating widespread immunization against cholera. The Pottevin–Madsen–White Commission also reported that the 80,000 Russian prisoners of war in Polish custody were not receiving adequate nutriture and clothing, with prisoners suffering from typhus, relapsing fever, and cholera [104]. In October 1920, Eustachy Sapieha, the Polish Minister for Foreign Affairs, sought the assistance of the LRCS to care for these prisoners [87]. Nevertheless, an estimated 16–20,000 prisoners of war died in Polish captivity from disease, malnutrition, and exposure [105].

By contrast, northwestern Poland and the Posen area were essentially free of the disease. Also, the incidence of typhus in the Polish Army was low. Pottevin, Madsen, and White penned a communique that was published in *Lancet* on 4 December of 1920 describing the abysmal living conditions of the populace and rampant infectious diseases. The Commission concluded that although the Poles had made progress in typhus control, the currently available resources were inadequate, and that there was an urgent need for the LoN to provide the necessary resources to terminate the epidemic [104].

Hermann Biggs, the Health Commissioner of New York State, replaced Strong as Medical Director of the LRCS in October 1920. Biggs had previous experience combating a typhus epidemic in New York City (NYC) in 1897 [106]. Biggs was succeeded by Prof. Charles-Edward A. Winslow of Yale University from April to September of 1921 [12].

In November 1920, Amie Gauthier of the French Sanitary Commission to Poland concluded that that the cordon sanitaire had failed and the work of the mobile disinfection teams was ineffective. He decried the deficiencies in manpower and matériel that were necessary to successfully execute the anti-typhus campaign [99]. Altogether in 1920, there were 168,097 cases of typhus registered in Poland with 22,575 deaths [84].

In December 1920 Drummond again sent an urgent request for countries to pony up two million pounds ($115 million) for Polish relief work, but these appeals fell on deaf ears. Both Czechoslovakia and Hungary were also suffering typhus outbreaks and had no funds to spare [42]. Although Germany was the nation most threatened by the Polish situation, it made any contribution contingent on the involvement of its experts in the campaign, a proposal unacceptable to the LoN [14].

The end of the Polish–Soviet War in October 1920 brought hope that social conditions in Poland would soon improve. In January 1921, the Polish Army began to demobilize. Nevertheless, the winter of 1920–1921 was marked by industrial stagnation, high unemployment, rampant inflation, and coal shortages. The redistribution of land to the peasantry was sluggishly proceeding. Multiple labor strikes occurred in the first 3 months of 1921 [83]. To add to Poland’s misfortune, in the winter of 1920–1921 an outbreak of dysentery erupted, with 64,000 cases and 10,000 deaths [107]. However, due to bountiful harvests and ARA assistance, the food situation had improved by this time [83].

## 8. 1921: The Funding and Implementation of the Epidemic Commission

In January 1921 an American journalist for the Saturday Evening Post, Kenneth Roberts, visited Poland to investigate the refugee situation. In the early 1920s, Warsaw had become a great emigration center; due to the dismal economic conditions, the Polish government was readily issuing Polish passports, in some cases even to those who were not Polish citizens. The Bolshevik invasion in the summer of 1920 triggered a torrent of migration out of Poland. In 1920–1921 the American consulate granted 150,000 visas to Polish citizens; at least 75% were granted to Jews. To leave Poland, the emigrants would typically travel 12 h by train from Warsaw to Danzig. There, an officer of the US Public Health Service supervised the delousing of potential immigrants to the US to ensure that they were safe for transit. A ship could depart for the USA only if these hygienic procedures were performed on each passenger. In Danzig, immigrants were met by members of the Hebrew Immigrant Aid Society and directed to the Troyl emigration facility, a prisoner-of-war camp that had been transformed into a delousing complex. The baggage and outerwear were placed in a room for fumigation with hydrogen cyanide or chlorine gases. Other clothing was treated with steam. Mayhem typically ensued when the disinfestation rooms were opened and the immigrants scrambled to find their meager worldly belongings. The men were required to have their hair clipped. The barber chairs were placed in pans of creosote, and the nits on the hair and stray lice fell to their death into the creosote. Many of the immigrants protested the delousing, claiming that they were not lousy, but a cursory inspection invariably proved otherwise. Many of the immigrants were suffering from vagabond’s disease, a pruritic skin condition resulting from chronic louse infestation. However, for women, the hair was not clipped, and, for some men, their beards were allowed to be retained for religious reasons. All of the immigrants received a hot shower with soap. The immigrants were charged 60 Deutschmarks (about one US dollar at that time) each for two delousings. The Hebrew Immigrant Aid Society subsidized those unable to pay. After the two delousings, the emigrants were either housed in extremely crowded conditions at the camp, or sent to a barracks in the city until their ship was ready to sail. In the camp, two or three families were often housed in a single small room. Despite these measures, the ships carrying immigrants that were deloused at the camp were often not granted clean bills of health to sail to the USA. Roberts was clearly hostile to the immigration of these impoverished masses of potentially infested and infected Eastern Europeans to the USA, and he called the situation “the menace of the filth peril” [108].

Although France was Poland’s greatest military and economic ally [97], it was slow to provide medical assistance [42]. Britain finally contributed £50,000 ($3.4 million) without preconditions, allowing the EC to send its first supplies to Poland in April 1921. The LoN Assembly agreed to contribute £220,000 ($14.9 million), and by September 1921, £126,000 ($8.5 million) had been sent to the EC. Lithuania, Romania, and Czechoslovakia all requested assistance, but the funds of the EC were insufficient to engage in relief work in those countries [42]. In desperation, Drummond contacted the Director General of the LRCS to request an urgent appeal for medical and sanitary supplies [14]. Drummond and the French Deputy Secretary General of the LoN Jean Monnet continued to pressure the French government to make a contribution. Finally, in December 1921, the French awarded 2.5 million francs ($3.5 million) to the EC, 18 months after Drummond’s original request [42].

In April 1921, an advisory board for the EC was created, consisting of Charles-Edward Winslow (LRCS), Edouard Frick and Dr. Frederic Ferriere (ICRC), Thorvald Madsen (OIHP Director), and Rachel Crowdy (LoN); its goal was to coordinate the activities of the major international health organizations. They visited Poland in April 1921 and were impressed by the work of the Typhus Commissariat. By this time the level of typhus had decreased 78% compared to April 1920, but it was still 4000% higher than the average annual cases in the period 1905–1911 [36]. After the advisory board visit, the EC committed to provide the necessary equipment for 50 50-bed hospitals in Poland [33].

Meanwhile, in March 1920, the Jewish Joint Distribution Committee (JJDC) appropriated $500,000 ($28.7 million) for a medical-sanitary mission to Eastern Europe and appointed Dr. Harry Plotz of Mt. Sinai Hospital in NYC to organize the medical unit [109]. (Plotz was well-regarded in medical circles at the time because he had reported that he had discovered the causative organism of typhus and he developed an anti-typhus vaccine, but these claims were refuted by 1921 [110]).

**Figure A9:**
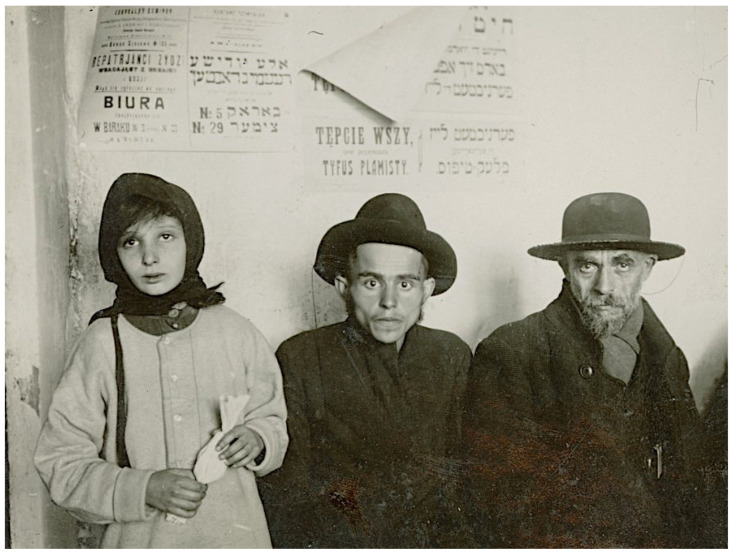
Original caption: “three starving Jewish refugees… in [a town] visited by … representatives [of] American. Jewish Joint Distribution Committee”. The poster behind the middle person refers to “exterminate lice (tepice wszy) {unreadable} spotted typhus (tyfus plamisty)” Rowne, Poland 1921 [149].

Plotz found that many of Poland’s 2.1 million Jews (about 8% of Poland’s population [111]) were living in squalid refugee camps (see Appendix A, Figure A9). Furthermore, anti-Semitic oppression was often disguised as measures against typhus, leading to violence, from beards being forcibly shaved to massacres. Many Jewish hospitals had been commandeered by the government and were being used as military hospitals. Early in 1921, Plotz was joined in Europe by a medical unit of 17 persons, later called the Medical Commission. The delegation distributed literature in Yiddish on typhus, opened baths, and distributed soap. Also, a film was produced and shown to the local populace about the transmission and prevention of typhus. Although initially the Polish government cooperated, Plotz faced many obstacles as local governments often reneged from their original support [112]. Plotz also acted as an advisor to the EC, but he resigned from the Medical Commission on 30 September 1921. The Medical Commission continued its activities from 1922 to 1923 under the direction of Dr. Jacob Golub [113].

**Figure 9:**
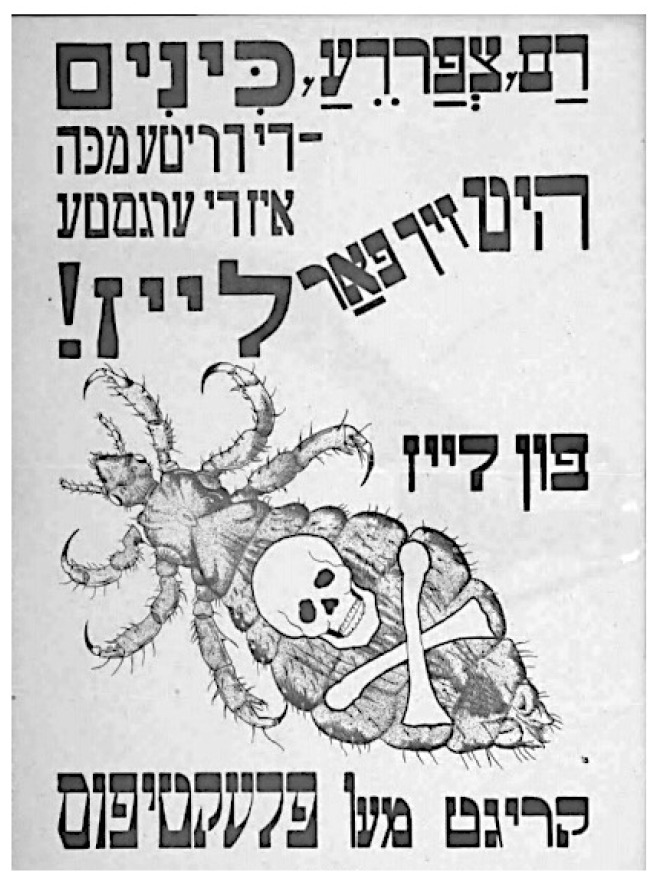
Poster in Yiddish issued by the Society for the Preservation of the Health of the Jewish Population [112]. The poster reads: “Blood, frogs, lice; The third plague is the worst; Protect yourself from lice!; From lice one contracts typhus” Poland, early 1920s.

Other anti-typhus activities amongst Polish Jews were conducted by the OZE (Obshchestvo Zdravookhraneniia Evreev; Society for the Preservation of the Health of the Jewish Population) [112]. The goals of OZE were to provide health and hygiene education, control contagious diseases, and improve maternal and child health [114]. Health education by the OZE was achieved through publications, publicity, and posters in Yiddish (Figure 9). Expositions and conferences were sponsored to provide information on hygiene and to combat superstitious and unhygienic customs among the Jewish populace [115].

**Figure 10:**
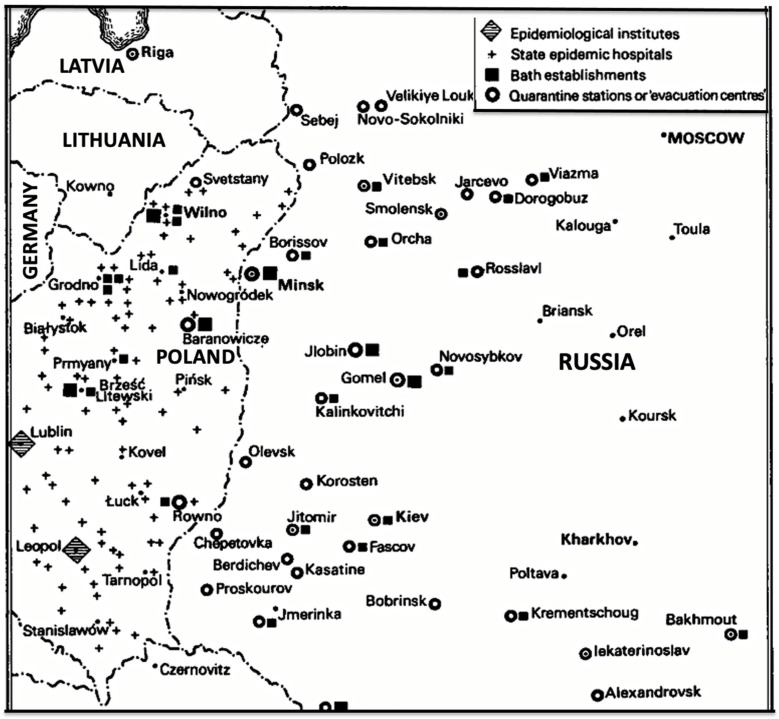
Map of epidemic hospitals, quarantine stations, and epidemiological institutes on the Polish–Russian frontier, published in 1922 [42].

In summer 1921, with modest funding in place, the EC set up their headquarters in the State Institute of Hygiene. With funding to the EC much less than expected, the priorities were: equipment for a 50-bed hospital; clothing for quarantine stations; the construction and repair of hospitals and bathing and disinfection facilities; and the acquisition of soap and medications. It was also necessary for the EC to focus its effort in a more restricted geographic area than originally planned, the Lida–Grodno–Białystok–Brześć Litewski line (Figure 10) [42].

The Peace of Riga, signed 18 March 1921, concluded the war between Poland and Soviet Russia. A separate treaty included specific provisions for the repatriation of prisoners of war. Seven ports of entry were established between Russia and Poland for the repatriation of Polish prisoners of war, with the largest two quarantine stations being at Baranowicze and Rowno; the former was larger, with one million entries in 1921 [84,116]. After the Polish–Soviet war, the ARC operated five hospital trains with 10 cars each that penetrated deep into the areas of rural eastern Poland that had been devastated by the recent war [117]. However, the efficient use of sanitary trains in Poland was hindered because of the different gauges of the tracks running northeast to southwest and those running northwest to southeast [35].

The number of typhus cases reported for January 1921 had fallen to 7000, versus 32,000 1 year earlier. By April, the Polish government had equipped 117 hospitals with 7625 beds and had 392 field disinfecting units operating in the most typhus-stricken areas, surpassing the medical and sanitary infrastructure that had been lost in the Polish–Soviet War. During the summer of 1921 the epidemic hospitals were largely occupied by malaria patients instead of those with typhus and relapsing fever [84]. Finally, clear progress in the battle against typhus in Poland was evident.

Nevertheless, it was apparent in 1921 that the cordon sanitaire established at key crossing points on the Polish–Russian frontier was failing to intercept a large number of refugees who were diffusing undetected through forested areas, the “green border” [42]. The areas around the eastern frontier cities of Novogrudok, Wilno, Brest-Litovsk, and Volhynia were the most severely affected by typhus. However, even interior cities, such as Warsaw and Bromberg, were also stricken [118]. Rajchman thereby conceived of a zone sanitaire, with intensified border control, systematic delousing of villages and cities in the most affected areas, and chains of sanitary centers and hospitals two, three, or more deep, to facilitate rapid detection, isolation, and treatment of cases and their contacts. Ultimately, the zone sanitaire spanned an area 1000 km in length and comprised 132 contagion hospitals and 20 sanitary centers, with a staff of 189 medical officers and 450 nurses. The largest sanitary center was at Baronowicze, which was completed in April 1921, with funds from the ARA, the Polish–American Committee for Help to Children, the Polish White and Red Cross Societies, and the Young Men’s Christian Association. About 3000 refugees arrived daily at Baronowicze, and the quarantine facilities there could accommodate up to 10,000 persons [42]. From March to December of 1921, 301,287 refugees passed through quarantine there [118].

The triage protocol for arriving trains at Baronowicze was to first send obviously ill passengers to hospitals. The remaining passengers were sent to “sorting centers” for medical inspection; those passing inspection were then directed to bathing and disinfection stations. Clothing and personal effects were also disinfected. The passengers received clean underclothing, a second medical inspection, and a smallpox vaccination. All passengers then had to undergo an obligatory quarantine period [42]. The conditions within the train cars entering the sanitary centers were often horrific, containing multiple corpses and teeming with lice [33]. Of 50,981 refugees quarantined at Baronowicze in October 1921, 804 required hospitalization and 497 died. In November, of the 59,843 new arrivals, there were 1406 deaths. In the quarantine center, despite appropriate precautions, 114 nurses and other personnel out of 400 employees fell ill with typhus during the same period [118]. In the first week of December 1921, 540 of the passengers processed in Baronowicze required hospitalization; 59 died by the time they arrived at the hospital, and another 120 persons died during the hospitalization [33]. In late 1921, cholera struck Ukraine, and this presented a further threat to Poland [118].

The pitiful condition of the arriving refugees prompted Polish officials to lodge formal complaints to the Soviet Union and the LoN [119]. In the repatriation agreement the maximum number of refugees to be sent from Russia to Poland was set at 4000 per day [33], but on some days the Soviets were sending up to quadruple that number. Furthermore, the Soviets expended no effort to segregate the sick from the healthy or to provide medical care [42].

**Figure 11:**
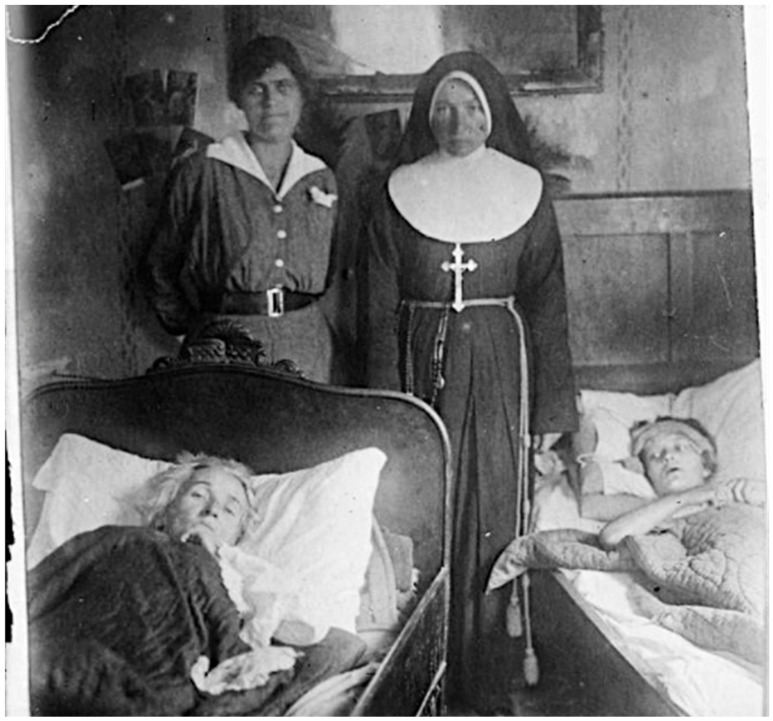
Original caption: “Mother and daughter ill with typhus in home at Sambor, Poland” [120]. 25 August 1921.

In August 1921, Rajchman was named the first director of the LoN Health Section, although he remained in Poland to facilitate the anti-typhus campaign until November. There was concern within the LNHO that without considering the health conditions in non-member states, it would be impossible to stem the tide of communicable diseases. An Epidemiologic Intelligence Service was created by the LoN in August 1921, which compiled statistics on typhus in Europe. In 1921, 44,835 cases of typhus were officially notified in Poland, a rate of 166/100,000 persons (Figure 11). Russia reported an incidence of 480/100,000, but this figure was considered to be a gross underestimate. The incidence rates per 100,000 in Latvia, Lithuania, Rumania, and Estonia were 179, 120, 43, and 20, respectively. By contrast, the typhus rate in Western Europe was only 1.5/100,000 [36]. Thus, Soviet Russia remained a source of typhus for neighboring countries [19]. With the continued flood of refugees into Poland and the ongoing typhus conflagration in Russia, it remained difficult to extinguish the Polish epidemic.

Unfortunately, in 1921, the campaign of the LRCS was faltering. First, the generosity of the public to fund the various Red Cross societies during World War I did not persist in peacetime [12]. Furthermore, in early 1921, the health of Henry Davison, the organization’s guiding light, was failing; plagued by debilitating headaches, he died of a brain tumor in May of 1922 [121].

**Figure 12:**
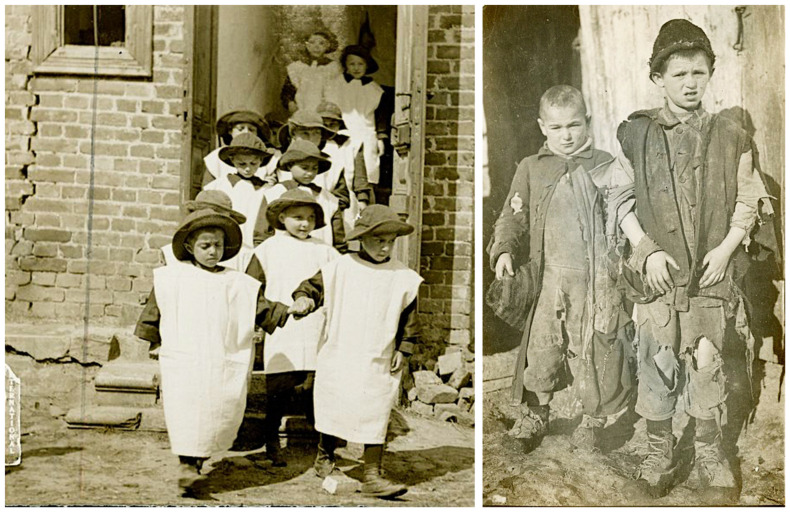
Examples of the “clothes famine” in post-war Poland. Original captions: **Left**: “Children outside an orphanage in post-war Grodno, using flour sacks as clothes (1920)” [123]. **Right**: “The two children…, dressed in rags, are orphans in Dubno… (1921–1922)” [124].

By late 1921, the food situation had improved in Eastern Europe, but now relief officials were faced with a “clothes famine” (Figure 12). In many areas, clothing had been difficult to obtain since 1915. In Poland, due to currency devaluation, in 1921 a new pair of shoes cost the same as a family’s food for a month [122]. The clothes famine was relevant to typhus control because during delousing operation there were no available spare clothes, and clothing infested with lice and their eggs and contaminated with infective louse feces was often scavenged from the dead and dying.

## 9. 1921: Soviet Cooperation with International Health Initiatives

In September 1921, the LNHO sent Rajchman and White to Russia to investigate its health conditions. The two men were granted an audience with Dr. Nikolai Semashko, the first Commissar for Health of Soviet Russia, and spent 6 days inspecting sanitary facilities and interviewing health workers [36,42]. Overall, their impression of the Soviet health infrastructure was favorable. Cholera, smallpox, dysentery, and plague were all in retreat. However, typhus and relapsing fever remained at epidemic levels, and malaria was problematic in the Volga River basin. Furthermore, poverty, famine, the lingering effects of an Allied economic boycott, a lack of medications, and widespread devastation from years of war and civil unrest all hindered further progress in disease control [36]. After the tour of Rajchman and White, Semashko agreed to bilateral sanitary agreements with Poland and to accept technical and material aid [22]. After the LNHO donated £3500 worth of drugs ($236,000), the Soviet government sent health statistics to the LoN headquarters and negotiated with the Polish government over mutual health concerns. This was especially important to Poland because assistance from the Rockefeller Foundation was contingent on a working relationship between Poland and Russia [22,36]. After the trip to Russia, the report of Rajchman and White convinced the LNHO of the need to bring sanitary activities into Russia [22].

In 1921, the LoN launched the publication Epidemiological Intelligence (EI). Six issues of EI were published between 1921 and 1922, all focusing on epidemics in Eastern Europe, including two issues (Epidemics in Russia since 1914, parts 1 and 2) authored by epidemiologist Lev Tarassevitch, the first director of the GINZ (Gosudarstvennyĭ nauchnyĭ institut narodnogo zdravookhranenii (the Soviet national biomedical research institute)). These reports provided the first glimpses into the epidemiology of infectious diseases in Russia since the ascension of the Bolsheviks [125].

The LNHO also started collaborating with Fridtjof Nansen, the High Commissioner of the ICRC-initiated Comité international de secours a la Russie [126]. At Nansen’s request for a medical expert, Rajchman appointed physician Reginald Farrar (formerly of the British Ministry of Health) as the LoN medical representative to Russia. The British Red Cross supported Nansen’s work with war surplus sanitary and medical supplies worth £100,000 ($6.85 million). Farrar was placed in charge of both the Sanitary Section of Nansen’s mission and the distribution of medical and sanitary supplies from the EC. He was expected to provide weekly reports about the infectious diseases’ situation in Russia. Farrar and Nansen surveyed the areas of Russia most afflicted by famine and pestilence, and they witnessed appalling conditions of widespread typhus and villages denuded of population by abandonment and the deaths of their inhabitants [42]. It was Rajchman’s hope that Farrar would serve as a permanent representative of the LoN Health Committee in Russia, but Farrar succumbed to typhus in December 1921 [127]. Meanwhile, famine descended upon Ukraine in November 1921, precipitating a new wave of refugees fleeing into Poland, with many filtering through the forested areas between the quarantine stations [33].

In February 1922, Rajchman persuaded the LoN Council to sponsor an international conference, citing the festering epidemic crisis in Russia, breaches in the Polish sanitary cordon, typhus and cholera outbreaks in Ukraine, and the invasion of typhus into Prussia [19]. From 20–28 March 1922, the European International Health Conference convened in Warsaw. All European nations were invited, including those that were not members of the LoN, such as Germany and Russia. Representatives from 27 nations attended. The USA was invited but declined to participate. Philanthropic organizations were not invited; by intentionally excluding them, Rajchman wanted this conference to induce governments to commit to cooperation and the funding of international health initiatives [14].

However, the Soviets mistrusted the LoN because it did not chastise Poland for its aggression against Russia and member nations of the League had supplied Poland with war matériel and advisors during the Polish–Soviet War [128]. Thus, initially the Russians refused to attend. Then they agreed to attend, but only if Russia was not held responsible for the current epidemic crisis. By the time of the meeting, they sent the largest delegation. The conference was opened by a speech of the Polish Minister of Foreign Affairs, Konstanty Skirmut, who stated that the rebuilding of war-torn Europe was impossible without arresting the ongoing epidemics. Rajchman then proceeded to give an overview of the typhus situation in Europe, expounding on the hazards of uncontrolled communicable diseases to the economic well-being of the continent [22].

The emphasis of the conference was typhus control in Russia. William Haigh, the LNHO representative to Russia, testified that the refugee situation in Ukraine and White Russia was out of control, with many of the refugees living in the forests, inaccessible to public health interventions. Haigh presented the urgent need for delousing and quarantine stations at the railway junctions of Minsk and Smolensk. He also criticized the inadequacy of disinfection procedures in Moscow as a factor in allowing infested refugees to proceed westward. The conferees concurred that epidemics require international cooperation for resolution [129].

The resolutions adopted at the conference included: bilateral sanitary agreements; mutual recognition of medical credentials to facilitate personnel exchanges; regulations on the notification of communicable diseases; the implementation of public health campaigns; and the provision for specialized training of medical personnel in Warsaw, Moscow, and Kharkov [42]. The conferees proposed a three-pronged public health intervention in Russia: intensification of the existing cordon sanitaire; an extension of the sanitary zone to the Black Sea; and applying basic sanitation measures into the Donbas, an economically vital area that was beset by famine and pestilence [14]. The German representatives at the conference were Gottfried Frey, Richard Otto, and Peter Mühlens, all infectious diseases experts. The Germans displayed great antipathy toward the LoN but agreed to the resolutions when they were promised a position in the LNHO. The French were incensed with German participation, the use of German (and not French) as one of the official languages and giving the Germans a seat in the LNHO [42].

Rajchman proposed continuing a sanitary cordon, comprised of a 1500 km chain of hospitals, quarantine stations, and delousing facilities, with Poland and Romania on one side and Russia and the Ukraine on the other, but this proposal was rejected by the assembled delegates [14]. Although the funding and political will for interventions in Russia never materialized, the meeting nonetheless was groundbreaking; it brought nations together that had been antagonistic, and it implemented concrete steps that transcended national borders to improve health. With this break in the ice, in 1923, Semashko requested medical and sanitary supplies from the LoN, due to shortages in the Soviet Union of medications, laboratory equipment, diphtheria antisera, and soap [129], even though the Soviet Union had not yet been permitted to join the LoN.

Concern about the Polish epidemic extended all the way to New York City (NYC). Royal Copeland, president of the NYC Board of Health, was apprehensive about the epidemics transpiring in Europe because NYC was the entry point of 87% of European immigrants to America at that time [130]. On 10 February 1921, Copeland telegraphed President Wilson imploring him to declare an immediate ban on immigration from typhus-ridden ports [131]. Copeland later dispatched an emissary to Poland, Col Edward Gibbs, to assess the overall situation. Gibbs returned to NYC in April 1922 and reported on the high rate of typhus among the refugees streaming into Poland, the shortage of physicians (only 3000 for a population of 30 million), the overcrowded hospitals, and the high mortality rate among the typhus sufferers [132]. Copeland himself visited Poland in 1922 and was provided with transportation and an interpreter and was given free rein to observe conditions in eastern Poland. After he returned to NYC in August 1922, Copeland reported that three of the seven entry points into Poland from Russia had no proper medical inspection and quarantine. Copeland erroneously proclaimed that the number of typhus deaths in Russia to be 5 to 45 million. Copeland declared that if typhus reached NYC, it would kill a million in a month and appealed to the citizens of NYC for $100,000 ($1.87 million) to construct a hospital in Poland [116]. In that same month, the LoN Health Committee announced that its funds to support the anti-typhus campaign in Poland were exhausted [42]. Fortunately, the epidemic was drawing to a close.

## 10. 1923–1924: The Epidemic Winds to a Close

In 1921, the number of typhus cases in Poland declined to 49,547; by 1923, cases dropped to 11,185. In 1924, the number of cases was 7706, approaching pre-World War I levels [11]. The Epidemic Commission helped maintain the sanitary cordon on the Russian border through 1924 [14]. After eight agonizing years (1916–1923) and over 400,000 documented cases and over 130,000 deaths (however, these figures do not include cases and deaths in Russian-occupied Poland from 1916–1918) [7], the Polish typhus epidemic was finally over. (Other estimates are four million cases from 1919–1921, with a 10% mortality rate) [40]. Although the threat of typhus to Western Europe may have been exaggerated to help secure aid to Poland for both humanitarian and geopolitical reasons (i.e., to prevent the spread of Bolshevism) [7], Poland had nonetheless suffered one of the greatest typhus epidemics of modern times.

Multiple factors led to the amelioration of the typhus situation in Poland over these 8 years. At this difficult juncture in its history, Poland was fortunate to have a physician-diplomat native son of considerable talents and energy working tirelessly on its behalf: Dr. Ludwik Rajchman. In addition to being fluent in Polish, English, and French, Rajchman had forged many scientific and political connections in France and England. With his scientific and medical credentials, multilingualism, and his network of colleagues abroad, Rajchman was ideally suited for securing international assistance for areas threatened by infectious diseases, despite the challenging political and economic circumstances of the time [22]. Also, the armed border disputes between Poland and its neighboring states (Russia, Lithuania, Germany, Ukraine, and Czechoslovakia) had finally ceased. In addition, the process of repatriation of prisoners of war and displaced civilians had concluded in 1923 [103]. Furthermore, there had been a slow but steady influx of sanitary, food, economic, and medical aid and expertise from various organizations to Poland since 1919, including the LRCS, American–Polish Relief Expedition, American Jewish Joint Distribution Committee, LoN Epidemic Commission, ARA, various Polish–American charities, Polish White and Red Cross Societies, Adam Sapieha’s Relief Committee, Young Men’s Christian Association, American and British Quakers, Save the Children Fund, General Committee of Assistance to the Victims of War in Poland, Polish–American Children‘s Relief, ARC, Polish Grey Samaritans, American Child Fund, Imperial War Relief Fund, and Vienna Relief Fund [14,23,42,133]. Administratively, within Poland, the anti-typhus campaign was also conducted more effectively by the Extraordinary Epidemic Commissariat [90,91,92,93,94]. Microbiologist Hans Zinsser gave the credit for taming typhus in Poland to the unremitting fortitude of the Polish people [134].

## 11. Typhus Relief to Poland in Perspective

The total aid provided to Poland from external sources from 1919 to 1922 has been estimated to be $193 million ($3.6 billion), with about $156 million ($2.9 billion) provided by the US Government [34]. Despite the magnitude of this sum, it was inadequate to promptly address the colossal task at hand [135].

After assisting during a refugee crisis in Greece in 1924, the EC was disbanded by the LoN, despite Rajchman’s objections. The EC was the victim of a lack of political will, uncertain funding, improving European health conditions, and changing paradigms of population health [14,19]. By this time, the quarantinist approach of the EC that emphasized disinfection, quarantine, and sanitary cordons had given way to the concept of social hygiene, which focused on the conditions that increased a population’s susceptibility to disease. This shift from quarantinism to social hygiene in the mid-1920s was propelled by two factors: the great epidemics of Russia and Eastern Europe had finally subsided, and the financial support of social hygiene programs by the Rockefeller Foundation, beginning in 1922 [19]. In 1924, the annual number of typhus cases in Russia (122,546) had essentially declined to their 1913 levels (118,419 cases) [136].

The formation of the LRCS and the EC came at time of biomedical history in which the etiologies and routes of transmission of many of the great infectious scourges of mankind were known with certainty, and thus the means to arrest contagion could be applied if the resources were made available [20]. Certainly, the work of the LRCS and the LoN in epidemic control in Eastern Europe in the post-war period was not an unqualified success. In August 1921 Rockefeller Foundation Director George Vincent opined to John D. Rockefeller Jr that the LRCS campaign had “accomplished practically nothing” [7]. Historian Patricia Sealey called the Epidemic Commission a “failed experiment” [14]. Clearly, the pace of assistance moved ponderously slow due to the lack of national commitments and the depressed economic climate of the post-war period. During the multipartite negotiations concerning the Polish relief effort, rivalry and animosity riddled the relationships between the various involved parties (i.e., France against Germany; ICRC vs. LRCS; Soviets vs. Western nations) [12,14,19,36]. To quote historian Patrick Zylberman, “despite the wave of epidemics, nation-states and people did not cooperate. The common danger did not arouse a joint defensive response, nor did the general feeling of mutual vulnerability spur a reaction of solidarity” [137]. (Unfortunately, the same statement could be applied to the COVID-19 pandemic in the United States in 2020–2022). Also, the typhus epidemics in Poland and Russia did not spur scientific progress in louse control or typhus treatment and vaccinology. Other than Polish biologist Rudolf Weigl’s louse gut vaccine [138], other advances against typhus were not achieved until the 1930s and 1940s [139].

Nevertheless, the material aid, expertise, and advocacy provided by the EC, the APRE, and the LRCS likely contributed to the resolution of the epidemic of louse-borne disease in Poland. This was the first test of international cooperation being applied to ameliorate a health problem; a new paradigm in international relations had thus been established, if not executed with full success [14]. Unfortunately, it would take another world war and the creation of the World Health Organization before more effective global cooperation for the betterment of populational health would emerge. Part II of this series will address the Ebola Virus Disease epidemic of West Africa in 2013–2016. In contrast to the Polish epidemic, the response to this epidemic was more prompt, and significant scientific progress was achieved within a few years after this epidemic.

## Appendix B. Image Credits and Sources

**Table:**

Figure No.	Left	Center	Right
Figure 1	-----	LoC (public domain)	-----
Figure 2	-----	LoC (public domian)	-----
Figure 3	-----	Permission granted, SCFHL	-----
Figure 4	-----	NMHM (public domain)	-----
Figure 5	-----	LoC (public domain)	-----
Figure 6	-----	LoC (public domain)	-----
Figure 7	-----	public domain, [69]	
Figure 8	-----	LoC (public domain)	
Figure 9	-----	AYIJR (public domain), [112]	-----
Figure 10	-----	public domain, [41]	-----
Figure 11	-----	LoC, public domain	-----
Figure 12	Permission granted, AJJDC	-----	Permission granted, AJJDC
Figure A1		AMDCHH (public domain)	
Figure A2		UN Archives (public domain)	
Figure A3	Permission granted, SCFHL	----	Permission granted, SCFHL
Figure A4		Permission granted; Hoover Institution Archives a	
Figure A5		LoC (public domain)	
Figure A6	USPHS (public domain)	Creative Commons Attribution 4.0 International license	Public domain
Figure A7		public domain	
Figure A8		NMHM (public domain)	
Figure A9		Permission granted, AJJDC	

^a^ Papers of Major General Harry L. Gilchrist. Abbreviations: AJJDC, American Jewish Joint Distribution Committee; AMDCHH, Army Medical Dept Center of History and Heritage; AYIJR, Archives of the YIVO Institute for Jewish Research; LoC, Library of Congress; NLM, United States National Library of Medicine; NMHM, National Museum of Health and Medicine; SCFHL, Swarthmore College Friends Historic Library; UN, United Nations.

## Appendix C. Quotes

**Quote 1.** Sidney Brooks, an economist of the American Relief Administration (ARA), recorded his observations during a journey through the hellscape of post-war Poland:

“… as one passed along the main roads leading out of Warsaw to the east could be seen masses of trees blasted by artillery fire, destroyed bridges barely replaced by rough wooden structures, frequently recurring military cemeteries, shallow trenches at close intervals, and then upon nearing the eastern frontier, damaged, destroyed, or utterly demolished houses in the midst of fields pock-marked by shell holes, until one arrived at the main section of devastation when all that met the eye was but a crumble of bricks, stones, shattered trees, and torn up fields. That was the desolation one saw along the road from Warsaw to Pinsk,… more than 200 miles… the poor people throughout a whole land wretchedly clothed in rags, emaciated, utter hopelessness staring from their eyes, amid unspeakable stench and the filthy surroundings could give the unforgiveable, indelible picture of Poland…” [85].

**Quote 2.** Major Willis Baker of the APRE described the conditions in one of the Polish hospitals in 1919:

“845 beds in hospital, mostly typhus, but also tuberculosis, dysentery, relapsing fever, and smallpox. An August (1919) convoy of 200 (patients) had 30 cases on arrival, with 150 more cases [of typhus] developing in a few days. The staff remained uninfected until December when 400 prisoners were passing through daily, of whom 80% were infected and then the disease then broke out among officers and, of eight doctors, seven got typhus. Patients are lying on the floor of board platforms; there are straw and paper blankets; no hospital clothing, no medicines, no instruments, no hospital linen or equipment, almost no lavatories or latrines. The whole place is foul and terrible. A great shortage of eating utensils; 800 simple pots to feed 800 patients, who are in the most extreme misery” [59].

**Quote 3.** In late 1920, in response to a request by the Polish Red Cross to assess the conditions after the Polish–Soviet War, the ICRC sent a fact-finding delegation to Poland, led by Lucien Brunel, its secretary.

Brunel visited these internment camps and described the pitiful conditions with respect to food shortages and disease.

"The food, which is provided by the Polish soldiers, would be enough if the Polish government distributed it regularly. Sugar, lard and flour were in short supply… The number of the ill is increasing every day and the 1300 hospital places are not enough to deal with all the sick resulting from typhus and dysentery. Most of them have chest infections and the number of deaths is increasing because of the season. Many of these wretched people will not make it through the winter…” [97].

## Appendix D. Highlights of the Film *Le CICR Lutte Contre Le Typhus en Pologne*, 1921 [100]

At 1:35, the film shows a louse defecating on an agar plate; likely, the louse was placed there in an attempt to culture the microbe causing typhus from the louse feces, because determining the etiology of typhus was an ongoing scientific quest at the time. There are also frames that show the guts dissected from the louse, likely to demonstrate the organisms causing typhus within the gut of the louse or for vaccine preparation [138,139]. At 3:00, a device for feeding lice on volunteers for vaccine preparation is shown. At 6:45, the washing of clothes is shown. At 6:50, clothes are shown on a rack (which would have been wheeled into a disinfection chamber). At 7:05, there is inspection of clothes for lice and their eggs. At 7:25, the shaving of hair to eliminate lice is demonstrated. The operation of a Thresh-type steam disinfector is shown at 8:53. At 10:19 some spray devices and flasks for the combustion of sulfur for disinfestation are shown being removed from a truck. The sprayers likely contained carbolic acid. There are also clips of an ICRC orphanage in Warsaw.

## References

[1] World Health Organization (2014). Ebola Virus Disease, West Africa—Update. https://www.who.int/emergencies/disease-outbreak-news/item/2014_07_01_ebola-en.

[2] Dixon M.G., Schafer I.J. (2014). Ebola Viral Disease Outbreak—West Africa, 2014. Morb. Mortal. Wkly. Rep..

[3] The Race to Control Ebola in Sierra Leone Doctors without Borders. https://www.doctorswithoutborders.org/latest/race-control-ebola-sierra-leone.

[4] WHO Ebola Response Roadmap. 28 August 2014. http://apps.who.int/iris/bitstream/10665/131596/1/EbolaResponseRoadmap.pdf.

[5] Ebola Response: Where Are We Now?. http://www.doctorswithoutborders.org/document/ebola-response-where-are-we-now.

[6] WHO Ebola Response Team (2016). After Ebola in West Africa—Unpredictable risks, preventable epidemics. N. Engl. J. Med..

[7] Weindling P. (2000). Epidemics and Genocide in Eastern Europe, 1890–1945.

[8] Dyer R.E. (1943). Typhus fever. Med. Clin. N. Am..

[9] Bechah Y., Paddock C.D., Capo C., Mege J.-L., Raoult D. (2010). Adipose tissue serves as a reservoir for recrudescent *Rickettsia prowazekii* infection in a mouse model. PLoS ONE.

[10] Warrell D.A. (2019). Louse-borne relapsing fever (*Borrelia recurrentis* infection). Epidemiol. Infect..

[11] Berner W. (2008). History of the control of acute infectious diseases in Poland after the World War I until the year 1924 (including big cities). Prz. Epidemiol..

[12] Howard-Jones N. (1978). International Public Health Between the Two World Wars: The Organizational Problems.

[13] League of Red Cross Societies (1919). Proceedings of the Medical Conference Held at the Invitation of the Committee of Red Cross Societies, Cannes, France, 1–11 April 1919.

[14] Sealey P.A. (2011). The League of Nations Health Organisation and the Evolution of Transnational Public Health. Ph.D. Thesis.

[15] Goodall E.W. (1920). Typhus fever in Poland, 1916–1919. Proc. R. Soc. Med..

[16] Blackburn C. (2014). The rebirth of Poland: American humanitarianism after the Great War. Stud. Hist..

[17] Liulevicius V.G. (2005). War Land on the Eastern Front: Culture, National Identity and German Occupation in World War I.

[18] Weindling P. (1997). Purity and epidemic danger in German occupied Poland during the First World War. Paedagog. Hist..

[19] Rotramel S.A. (2010). International Health, European Reconciliation, and German Foreign Policy After the First World War, 1919–1927. Ph.D. Thesis.

[20] Dubin M.D., Weindling P. (1995). The League of Nations Health Organization. International Health Organisations and Movements, 1918–1939.

[21] Aldcroft D.H. (2018). Studies in the Interwar European Economy.

[22] Balińska M.A. (1998). For the Good of Humanity: Ludwik Rajchman, Medical Statesman.

[23] Thackeray F.W. (1990). To serve the cause of Poland: The Polish Grey Samaritans. Pol. Rev..

[24] Cornebise A.E. (1982). Typhus and Doughboys: The American Polish Typhus Relief Expedition, 1919–1921.

[25] Fisher H.H. (1928). America and the New Poland.

[26] Library of Congress Types of Under Nourished Children Received at the Refugee Station at Rowno. https://www.loc.gov/item/2017670257/.

[27] CPI Inflation Calculator. https://www.in2013dollars.com/us/inflation/1920.

[28] Measuring Worth. https://www.measuringworth.com/datasets/exchangepound/result.php?year_source=1900&year_result=2012.

[29] Hoover H. (1951). The Memoirs of Herbert Hoover. Years of Adventure 1874–1920.

[30] Hoover Institution (2005). An American Friendship: Herbert Hoover and Poland. https://www.hoover.org/events/american-friendship-herbert-hoover-and-poland.

[31] Fosdick R.B. (1920). The League of Nations at Work Without Us. New York Times.

[32] Strong R.P. (1920). The anti-typhus campaign in 1915 in Serbia considered in connection with the present typhus epidemic in Poland. Int. J. Public Health.

[33] Hutt C.W. (1927). International Hygiene.

[34] Adams M.L. (2017). Cadillacs to Kiev: The American Relief Administration in Poland 1919–1922.

[35] Gillett M.C. (2009). The Army Medical Department, 1917–1941.

[36] Borowy I. (2009). Coming to Terms with World Health: The League of Nations Health Organization 1921–1946.

[37] Durand A. (1984). History of the International Committee of the Red Cross from Sarajevo to Hiroshima.

[38] Foster G.M. (1982). The Demands of Humanity: Army Medical Disaster Relief.

[39] Library of Congress Freight Trains Crammed with Refugees. https://picryl.com/media/freight-trains-crammed-with-refugees-returning-to-their-homes-in-poland-after-e87b1e.

[40] Balińska M.A. (1996). The National Institute of Hygiene and Public Health in Poland 1918–1939. Soc. Hist. Med..

[41] Kreuder-Sonnen K. (2019). Epidemiological state-building in interwar Poland: Discourses and paper technologies. Sci. Context.

[42] Balińska M.A., Weindling P. (1995). Assistance and not mere relief: The Epidemic Commission of the League of Nations, 1920–1923. International Health Organisations and Movements 1918–1939.

[43] Przeniosło M., Przeniosło M. (2018). Polish medicine in 1918. Pol. Arch. Intern. Med..

[44] Towers B., Weindling P. (1995). Red Cross organizational politics. International Health Organisations and Movements 1918–1939.

[45] Back L.S. (2012). The Quaker mission in Poland: Relief, reconstruction, and religion. Quaker Hist..

[46] de Vries S. (2009). Blue Ribbons Bitter Bread: Joice Loch—Australia’s Most Heroic Woman.

[47] American Friends Service Committee Swarthmore College Special Collections. The Unit Delousing Whole Families. http://triptych.brynmawr.edu/cdm/singleitem/collection/SC_Relief/id/2183/rec/56.

[48] Wieckowska E. (1999). Fight against acute infectious diseases in Poland in the first year of independence (1918–1919). Prz. Epidemiol..

[49] (1919). Typhus in Europe a world problem. New York Times.

[50] Rolleston J.D. (1921). Typhus. Medical Science Abstracts and Reviews.

[51] Vermin in Cast-off Clothing Spreads Typhus in Holland. https://news.google.com/newspapers?nid=1126&dat=19190305&id=cVhRAAAAIBAJ&sjid=BWgDAAAAIBAJ&pg=1915,3541173)cVhRAAAAIBAJ&sjid=BWgDAAAAIBAJ&pg=1915,3541173.

[52] Stouman K. (1920). Invisible frontiers. Bull. Leag. Red Cross Soc..

[53] Moulin A.M., Weindling P. (1995). The Pasteur Institute between the two world wars. The transformation of the international sanitary order. International Health Organisations and Movements, 1918–1939.

[54] Churchill W. (1929). World Crisis. Volume IV: The Aftermath.

[55] The Sunday Times (1920). Parlous Poland. Ravaged by Bolshevik Typhus. http://trove.nla.gov.au/ndp/del/article/57971499.

[56] Hoover H. (1992). The Ordeal of Woodrow Wilson.

[57] Gilchrist H.L. (1920). Typhus fever in Poland. Milit. Surg..

[58] Gilchrist H.L. Report to Surgeon General, US Army. 1 February 1920. Archives of the American Jewish Joint Distribution Committee. http://search.archives.jdc.org/multimedia/Documents/NY_AR1921/00019/NY_AR1921_00688.pdf.

[59] (1920). Typhus in Poland. Lancet.

[60] MacKenzie M.D. (1941). Some practical considerations in the control of louse-borne typhus fever in Great Britain, in the light of experience in Russia, Poland, Rumania, and China. Proc. R. Soc. Med..

[61] Rhode M. (2008). Today’s discoveries. A Repository for Bottled Monsters. http://bottledmonsters.blogspot.com/2008/11/todays-discoveries.html.

[62] Foster G.M. (1981). Typhus disaster in the wake of war. The American-Polish Typhus Relief Expedition, 1919–1920. Bull. Hist. Med..

[63] (1919). Inter-Allied health commission to Poland. Lancet.

[64] Library of Congress (1919). The Interallied Medical Commission Sent by the League of Red Cross Societies to Investigate the Typhus Situation. https://www.loc.gov/item/2017670260/.

[65] Library of Congress (1919). A Typical Instance of the Scourge of Typhus Found by the Interallied Medical Commission. https://www.loc.gov/resource/anrc.04319/.

[66] (1920). All Poland ravaged by typhus epidemic; American experts report 95% of the people victims of disease. New York Times.

[67] (1920). Red Cross aid for Poland. New York Times.

[68] (1920). Items. Bull. Leag. Red Cross Soc..

[69] Wolbach S.B., Todd J.L., Palfrey F.W. (1922). The Etiology and Pathology of Typhus: Being the Main Report of the Typhus Commission of the League of Red Cross Societies to Poland.

[70] Palfrey F.W. (1922). The work of the League of Red Cross Societies’ Typhus Research Commission to Poland. Am. J. Public Health.

[71] Wolbach S.B. (1916). The etiology of Rocky Mountain Spotted Fever (a preliminary report). J. Med. Res..

[72] Wolbach S.B., Todd J.L. (1920). Note sur l’étiologie et l’anatomie pathologique du typhus exanthématique au Mexique. Ann. Lnst. Pasteur.

[73] Bacot A. (1921). On the probable identity of *Rickettsia pediculi* with *Rickettsia quintana*. Br. Med. J..

[74] da Rocha Lima H. (1926). Zur Aetiologie des Fleckfiebers. Berl. Klin. Wochnschr..

[75] Palfrey F.W., Wolbach S.B. (1921). Typhus fever. Med. Clin. N. Am..

[76] (1922). Typhus fever. A study of disease and its martyrs. JAMA.

[77] Military Times Hall of Valor Edward C. Register. https://valor.militarytimes.com/recipient/recipient-16787/.

[78] National Library of Medicine Images from the History of Medicine. Polish Typhus Relief Commission: Typhus ward in Grace Hospital, Warsaw, Poland. http://resource.nlm.nih.gov/101395239.

[79] James E.L. (1920). League prepares to succor Europe. New York Times.

[80] Goldman J. Letter from European Director General to Harry L. Gilchrist. https://search.archives.jdc.org/pdf_viewer.asp?lang=ENG&dlang=ENG&module=search&page=pdf_viewer&rsvr=ADMIN@ADMIN&param=%3Cwords%3ELetter!35;@from!35;@European!35;@Director!35;@General!35;@to!35;@Harry!35;@L.!35;@Gilchrist%3C/%3E%3Cpdf_path%3Emultimedia/Documents/NY_AR1921/00019/NY_AR1921_00605.pdf%3C/%3E%3Cbook_id%3E227822%3C/%3E&param2=&site=ideaalm.

[81] (1920). Typhus peril in Poland. American Jewish Relief Funds give $100,000 to help fight it. New York Times.

[82] Morris C.D. (1920). The American Red Cross in Europe. Bull. Leag. Red Cross Soc..

[83] Davies N. (2003). White Eagle, Red Star. The Polish-Soviet War 1919–1920 and ‘the Miracle on the Vistula’.

[84] Winslow C.E.A. (1922). European health conditions. Am. J. Public Health.

[85] Blackburn C. (2021). When typhus rode a red horse: Weaponizing disease during the Polish-Bolshevik War. Prz. Hist. Wojsk..

[86] Housden M. (2014). The League of Nations and the organization of Peace.

[87] (1920). League of Red Cross Societies and campaign against typhus in Poland. Bull. Leag. Red Cross Soc..

[88] (1920). American anti-typhus work in Poland. Lancet.

[89] News of the Red Cross Societies (1920). Poland. Bull. Leag. Red Cross Soc..

[90] Jaszczyński W., Great Flood Reflections of the Main Sanitary Inspectorate. http://www.racjonalista.pl/kk.php/s,3124.

[91] Wieckowska E. (1998). Central anti-typhus committee (1 August 1919–5 March 1920). Prz. Epidemiol..

[92] Perkowska U. (2002). The social activity of Professor Emil Godlewski Jr. Arch. Hist. Filoz. Med..

[93] Sliwa L. (2008). Emil Godlewski, Jr. (1875–1944) pioneer of embryology at the Jagiellonian University of Krakow (Poland). Int. J. Dev. Biol..

[94] Wieckowska E. (1999). The epidemic hospitals in Poland ordered or inspected by Chief extraordinary Epidemic Commissariat to fight against the epidemics (1920–1924). Arch. Hist. Filoz. Med..

[95] Clements K. (2010). The Life of Herbert Hoover: Imperfect Visionary, 1918–1928.

[96] Karpus Z. (2020). Soviet Prisoners of War 1919–1921. Institute of National Remembrance. https://eng.ipn.gov.pl/en/digital-resources/articles/4442,Soviet-prisoners-of-war-19191921.html.

[97] Healy J. (2003). Central Europe in Flux: Germany, Poland, and Ukraine, 1918–1922. Ph.D. Thesis.

[98] (1920). Typhus westward march fought. Am. Red Cross.

[99] (1920). Europe’s health frontier. Am. Med..

[100] International Committee of the Red Cross (2015). Lutte Contre le Typhus: Le CICR en Pologna, 1921. https://www.icrc.org/en/document/battling-typhus-icrc-poland-1921.

[101] Internet Encyclopedia of Ukraine Army of the Ukrainian National Republic. Bottom of Form. https://www.encyclopediaofukraine.com/display.asp?linkpath=pages%5CA%5CR%5CArmyoftheUkrainianNationalRepublic.htm.

[102] Balfour A.J. (1920). Appeal from the League of Nations. Bull. League Red Cross Soc..

[103] Housden M. (2007). When the Baltic Sea was a ‘bridge’ for humanitarian action: The League of Nations, the Red Cross, and the repatriation of prisoners of war between Russia and Central Europe, 1920–1922. J. Baltic Studies.

[104] Pottevin D., Madsen T., White R.N. (1920). Typhus and cholera in Poland: The action of the League of Nations. Lancet.

[105] Archiwa Panstwowe Polish-Russian Arrangements Regarding the Fate of Red Army in Polish Captivity. https://web.archive.org/web/20090114183643/http://www.archiwa.gov.pl/?CIDA=501.

[106] (1920). Appointments. Bull. Leag. Red Cross Soc..

[107] Sztuka-Polińska U. (2002). Epidemiological situation of the selected infectious diseases in Poland in 1918–1939. Prz. Epidemiol..

[108] Roberts K.L. (1922). Why Europe Leaves Home.

[109] Zalashik R., Patt A., Grossman A., Lvi L.G., Mandel M.S. (2019). Medical welfare in interwar Europe: The collaboration between JDC and OZE-TOZ organizations. The JDC at 100. A Century of Humanitarianism.

[110] Olitsky P.K. (1921). Experimental studies on the etiology of typhus fever. I. Concurrent infections during the course of experimental typhus fever in guinea pigs. J. Exp. Med..

[111] Laurentiu I. A study of the inter-war economies of Poland and Romania. Proceedings of the 7th International Days of Statistics and Economics.

[112] Davidovitch N., Zalashik R. (2008). «Air, sun, water»: Ideology and activities of OZE (Society for the preservation of the health of the Jewish population) during the interwar period. Dynamis.

[113] (1953). Dr. Jacob Golub, Noted Jewish Medical Director, Dies in New York. Jewish Telegraphic Agency. http://www.jta.org/1953/09/24/archive/dr-jacob-golub-noted-jewish-medical-director-dies-in-new-york#ixzz2f83OVYs3.

[114] Shvarts S., Romem P., Romem Y., Shani M. (2013). The mass campaign to eradicate ringworm among the Jewish community in Eastern Europe, 1921–1938. Am. J. Publ. Health.

[115] Rubenstein J. (1934). Protecting the health of Jews abroad. Jewish Soc. Service Quart..

[116] (1922). Dr. Copeland Warns of Typhus Failure. https://www.nytimes.com/1922/08/27/archives/dr-copeland-warns-of-typhus-peril-fears-epidemic-in-poland-which.html.

[117] (1920). Fighting typhus at the source. Pract. Med. Surg..

[118] (1922). Danger of an invasion by typhus fever. JAMA.

[119] Lenart F. (1922). Fear of invasion by typhus carriers. Am. J. Clin. Med..

[120] American National Red Cross Photograph Collection (Library of Congress) (1921). A Mother and Daughter Ill with Typhus. https://www.loc.gov/item/2017679378/.

[121] Lamont T.W. (1933). Henry P. Davison. The Record of a Useful Life.

[122] (1921). Red Cross News. Bull. Leag. Red Cross Soc..

[123] American Jewish Joint Distribution Committee Children Wearing Makeshift Coats from Flour Sacks Outside an Orphanage in Grodno, Poland. https://www.wdl.org/en/item/17327/.

[124] American Jewish Joint Distribution Committee (1920). Jewish War Orphans in Dubno, Poland. 1921–1922..

[125] Cliff A., Haggett P., Smallman-Raynor M. (1998). Deciphering Global Epidemics: Analytical Approaches to the Disease Records of World Cities, 1888–1912.

[126] Vogt C.-E. (2009). Fridtjof Nansen and European food aid to Russia and the Ukraine 1921-1923. Dvacáté Století.

[127] (1922). Typhus kills League agent. New York Times.

[128] (1920). Chicherin and the League of Nations. Sov. Russ..

[129] Weindling P., Solomon S.G. (2006). German overtures to Russia, 1919–1925: Between racial expansion and national coexistence. Doing Medicine Together: Germany and Russia Between the Wars.

[130] Typhus Wave (1922). Europe in Danger. Horrors in Poland. http://trove.nla.gov.au/ndp/del/article/66727873.

[131] Cabaniss E.R., Cameron A.E. (2017). ‘Unassimilable and undesirable’: News elites’ discursive construction of the American immigrant during the Ellis Island years. Discourse Soc..

[132] (1922). Colonel Gibbs warns of danger of typhus. New York Times.

[133] van Norman L.E. (1921). Danzig traffic for Poland. Commerce Rep..

[134] Zinsser H. (1940). As I Remember Him. The Biography of R.S..

[135] Forbes J. (1962). The Quaker Star Under Seven Flags, 1917–1927.

[136] Haines A.J. (1928). Health Work in Soviet Russia.

[137] Zylberman P., Bashford A. (2006). Civilizing the state: Borders, weak states and international health in modern Europe. Medicine at the Border.

[138] Polak A., Pawlikowska-Łagód K., Zagaja A., Grzybowski A. (2022). Typhus works of Rudolf Weigl, PhD, Ludwik Fleck, MD, and Eugeniusz Lazowski, MD, against the Nazis. Clin. Dermatol..

[139] Anstead G.M. (2018). Triumph over Typhus in World War II. https://pitt.hosted.panopto.com/Panopto/Pages/Embed.aspx?id=26969339-8557-4e42-ad62-a893013b24b2.

[140] Gillett M.C. (1995). The Army Medical Department, 1865–1917.

[141] American Friends Service Committee Swarthmore College Special Collections. Shelter for Widow and Children. http://triptych.brynmawr.edu/cdm/singleitem/collection/SC_Relief/id/2217/rec/6.

[142] American Friends Service Committee (1919). Poor Family’s Hut. Poland. http://triptych.brynmawr.edu/cdm/singleitem/collection/SC_Relief/id/2225/rec/16.

[143] (2019). Hoover Acquires the Papers of Major General Harry L. Gilchrist. https://www.hoover.org/research/hoover-acquires-papers-major-general-harry-l-gilchrist.

[144] Library of Congress (1919). Interallied Medical Commission. https://www.loc.gov/item/2017670252/.

[145] Woodward T.E., Walker D.H., Dumler J.S. (1992). The remarkable contributions of S. Burt Wolbach on rickettsial vasculitis updated. Trans. Am. Clin. Climatol. Assoc..

[146] John Lancelot Todd. https://commons.wikimedia.org/wiki/File:John_Lancelot_Todd_1905.jpg.

[147] Greenwood M., Arkwright J.A. (1924). The life and scientific work of Arthur William Bacot. J. Hyg..

[148] Bystrycka O. (2023). Tadeusz and Emil Godlewscy: Scientific Activities of Scientific Brothers. https://krakow1.one/pl/eternal/tadeusz-i-emil-godlewscy-dzialalnosc-naukowa-braci-naukowcow-3815.

[149] American Jewish Joint Distribution Committee Jewish Refugees in Rowne, Poland. https://www.wdl.org/en/item/17344/.

